# Distribution of *LGR5* ^+^ Cells and Associated Implications during the Early Stage of Gastric Tumorigenesis

**DOI:** 10.1371/journal.pone.0082390

**Published:** 2013-12-10

**Authors:** Bo Gun Jang, Byung Lan Lee, Woo Ho Kim

**Affiliations:** 1 Department of Pathology, Seoul National University College of Medicine, Jongno-gu, Seoul, Korea; 2 Department of Anatomy, Seoul National University College of Medicine, Jongno-gu, Seoul, Korea; University Claude Bernard Lyon 1, France

## Abstract

*Lgr5* was identified as a promising gastrointestinal tract stem cell marker in mice. Lineage tracing indicates that *Lgr5* **^+^** cells may not only be the cells responsible for the origin of tumors; they may also be the so-called cancer stem cells. In the present study, we investigated the presence of *Lgr5* **^+^** cells and their biological significance in normal human gastric mucosa and gastric tumors. RNAscope, a newly developed RNA *in situ* hybridization technique, specifically labeled *Lgr5* **^+^** cells at the basal glands of the gastric antrum. Notably, the number of *Lgr5* **^+^** cells was remarkably increased in intestinal metaplasia. In total, 76% of gastric adenomas and 43% of early gastric carcinomas were positive for *LGR5*. *Lgr5* **^+^** cells were found more frequently in low-grade tumors with active Wnt signaling and an intestinal gland type, suggesting that *LGR5* is likely involved in the very early stages of Wnt-driven tumorigenesis in the stomach. Interestingly, similar to stem cells in normal tissues, *Lgr5* **^+^** cells were often restricted to the base of the tumor glands, and such *Lgr5* **^+^** restriction was associated with high levels of intestinal stem cell markers such as *EPHB2*, *OLFM4*, and *ASCL2*. Thus, our findings show that *Lgr5* **^+^** cells are present at the base of the antral glands in the human stomach and that this cell population significantly expands in intestinal metaplasias. Furthermore, *Lgr5* **^+^** cells are seen in a large number of gastric tumors ; their frequent basal arrangements and coexpression of ISC markers support the idea that *Lgr5* **^+^** cells act as stem cells during the early stage of intestinal-type gastric tumorigenesis.

## Introduction

Cancer stem cell theory has drawn considerable attention since it was first demonstrated in hematologic malignancies. A growing body of evidence from studies of various solid tumors also supports the concept [1]. Because cancer stem cells (CSCs) are believed to be the only tumor cell subpopulation with the potential to establish an entire tumor, especially after chemoradiation therapy, CSC targeting may be a novel approach through which to improve patient outcome [2]. Although many proteins have been proposed as markers of cancer stem cells, the identification and confirmation of such markers in normal human tissues remains challenging. In the gastrointestinal tract, a number of putative stem cell markers such as *Lgr5* [3], *Bmi-1* [4], and *Prominin-1* [5] have been identified. Among these, *Lgr5* (*Leucine-rich repeat containing G-protein-coupled receptor 5*) is the most promising and established marker. Lineage tracing experiments have shown that *Lgr5* is an adult stem cell marker expressed in the small intestine, colon, stomach, and hair follicles in mice [3]. *Apc*-mutant *Lgr5* **^+^** cells were reported to be the origin of progressively growing adenomas [6]. Additionally, *Lgr5* **^+^** cells were found to be the multipotent stem cells that produced all other adenoma cell types in intestinal adenomas [7]. *Lgr5* seems to be the first reported biomarker for stem cells in both normal intestinal mucosa and corresponding tumor tissues.

For several decades, the isthmus region of the stomach has been widely accepted as a stem cell reservoir, based on indirect evidence such as a high proliferative activity and the presence of immature granule-free cells that resemble embryonic stem cells [8]. However, *in vivo* lineage tracing revealed that a group of *Lgr5^+^* cells at the base of the pyloric glands were multipotent stem cells that contributed to daily epithelial renewal [9]. The Wnt-driven tumor initiation induced by targeted ablation of *Apc* tumor suppressor activity was also suspected to occur in the stomach *LGR5* ^+^ cells. [9]. Despite the discoveries pertaining to *Lgr5* as an adult stem cell marker in mice, the relevance of *Lgr5* expression in human tissues has not been fully evaluated. This is largely because the *in vivo* lineage tracing technique, which was used in mice to demonstrate the stem cell activity of candidate cells, cannot be applied to human stem cell population studies [8]. Although several studies have attempted to determine the presence of *Lgr5* **^+^** cells either with antibodies [10]-[13] or with RNA *in situ* hybridization (ISH) [14], [15], none of the studies provided convincing evidence supporting the presence of *Lgr5* **^+^** cells in human tissues. Indeed, the lack of a reliable antibody to LGR5 is the main obstacle in identifying human counterparts of mouse *Lgr5^+^* cells for use in clinical applications.

In the present study, we show that *Lgr5* **^+^** cells are exclusively located at the base of the antral glands in the human stomach and that this cell population is remarkably expanded in intestinal metaplasias (IMs). This expansion may be a critical factor in the development of tumors from IMs. Most gastric adenomas (GAs) contain a number of *LGR5*-expressing tumor cells that typically reside at the basal areas of tumor glands in a similar manner to *Lgr5* **^+^** cells in the intestinal mucosa, as well as in IMs. Furthermore, the lower halves of GA glands, which harbor most of the *Lgr5* **^+^** cells, specifically coexpress intestinal stem cell markers such as *EPHB2*, *OLFM4*, and *ASCL2,* as well as *CD133*, thus supporting the hypothesis that *LGR5* is a tumor stem cell marker during the early stage of intestinal-type gastric tumorigenesis.

## Materials and Methods

### Subjects

We analyzed formalin-fixed and paraffin-embedded (FFPE) gastric tumors collected from 159 patients who underwent endoscopic submucosal dissection (ESD) at Seoul National University Hospital, Seoul, Korea, from 2008 to 2010. Clinicopathological data such as patient age and gender, histological tumor type, Lauren’s classification, and evidence of lymphatic invasion were obtained by reviewing the medical charts and pathological records. A normal human skin specimen, including hair follicles, was obtained from a patient with basal cell carcinoma who underwent surgery, and normal small and large intestine samples, which were confirmed to be normal, non-cancerous tissues by histopathological analyses, were obtained from a patient with colon cancer who underwent a colectomy. Unfixed, fresh-frozen, normal gastric tissues were available from 11 patients with gastric cancer who underwent gastrectomy from 2001 to 2005 at Seoul National University Hospital.

### Ethical statement

All human specimens were obtained during surgery. The participants did not provide written consent to participate in this study. The retrospective study was performed using the stored samples after the pathologic diagnosis, and all of the samples were anonymized before the study. This retrospective study design was approved by the Institutional Review Board at Seoul National University Hospital under the condition of anonymization (reference: H-1209-037-424).

### Tissue microarray (TMA) construction

Core tissue biopsies (2 mm in diameter) were obtained from individual FFPE gastric tumors (donor blocks) and arranged in a new recipient paraffin block (tissue array block) using a trephine apparatus (SuperBioChips Laboratories, Seoul, Korea). Three TMAs were produced, each of which contained 53 gastric tumors that had been removed by ESD, and 7 normal non-tumorous gastric mucosa samples, including the antral glands, fundic glands, and IM. An additional TMA, comprising 30 active gastritis cases, was also constructed from the specimens of the patients with gastric tumors.

### RNA *in situ* hybridization (ISH)

ISH for *LGR5*, *EPHB2*, *ASCL2*, *OLFM4,* and *CDX2* was performed with the RNAscope FFPE assay kit (Advanced Cell Diagnostics, Inc., Hayward, CA, USA) according to the manufacturer’s instructions. Briefly, 4 µm formalin-fixed, paraffin-embedded tissue sections or TMA sections were pretreated with heat and protease digestion and then hybridized with a target probe for *LGR5*. Thereafter, an HRP-based signal amplification system was hybridized to the target probe before color development with 3,3′-diaminobenzeidine tetrahydrochloride (DAB). Positive staining was defined as the presence of brown punctate dots in the nucleus and/or cytoplasm. The housekeeping gene ubiquitin C (UBC) served as a positive control. The *DapB* gene, which is derived from a bacterial gene sequence, was used as a negative control. For gastric tumors, *LGR5* staining was graded based on the percentage of tumor cells that expressed *LGR5* as follows: grade 0, absence of *Lgr5^+^* tumor cells; grade 1, 1%–5% of *Lgr5^+^* tumor cells; grade 2, 6%–25% of *Lgr5^+^* tumor cells; and grade 3, 26%–100% of *Lgr5^+^* tumor cells. The results were grouped as positive (grade 2 or 3) or negative (grade 0 or 1), given that normal gastric mucosa was identified as grade 1 for *LGR5* expression.

### Immunohistochemistry

Immunohistochemistry was performed on 4 µm TMA sections using a BOND-MAX automated immunostainer and a Bond Polymer Refine Detection kit (Leica Microsystems, Wetzlar, Germany) according to the manufacturer’s instructions. The Ventana BenchMark XT automated staining system (Ventana Medical Systems, Tucson, AZ, USA) was only used for claudin-18 staining. The primary antibodies used were anti-β-catenin (Novocastra Laboratories Ltd., Newcastle, UK; 17C2; 1∶800), anti-CD10 (Novocastra; 56C6; 1∶100), anti-CDX2 (BioGenex, San Ramon, CA, USA; CDX2-88; 1∶500), anti-MUC2 (Novocastra; Ccp58; 1∶300), anti-MUC5AC (Novocastra; CLH2; 1∶300), anti-MUC6 (Novocastra; CLH5; 1∶100), and anti-claudin-18 (Invitrogen, Carlsbad, CA, USA; 34H14L15; 1∶1000) antibodies. Nuclear β-catenin staining was considered positive when more than 10% of the tumor cell nuclei were strongly stained for β-catenin. MUC5AC is expressed in foveolar cells in the stomach, and MUC6 is expressed in mucous cells in the neck of the oxyntic mucosa or in the pyloric glands. MUC2 is expressed in goblet cells with IM in the stomach. CD10 glycoprotein is expressed on the brush borders of intestinal epithelial cells. MUC5AC and MUC6 are gastric phenotypic markers, and MUC2 and CD10 are intestinal phenotypic markers [16]. Based on the phenotypic combinations of mucin expression, gastric tumors were classified into the following 4 groups: combined, gastric, intestinal, and unclassified [17].

### Laser-capture microdissection and RNA extraction

For each patient, the upper and lower portions of the tumor glands and the normal gastric and intestinal mucosa were isolated from 5 to 6 sections. Briefly, 4 µm paraffin-embedded tissue sections were obtained, and the areas of interest were selectively microdissected using a laser microdissection device (ION LMD, Jung Woo International Co., Seoul, Korea) without deparaffinization or staining procedures in order to minimize further cellular RNA damage. Total RNA was extracted from the laser-captured areas with an RNeasy FFPE Kit (Qiagen, Valencia, CA, USA) according to manufacturer’s instructions, with a slight modification involving extended proteinase K digestion for at least 17 hours after the deparaffinization step [18].

### Quantitative real-time PCR

cDNA was prepared from 0.5–1 µg of total RNA with random hexamer primers and the GoScript reverse transcription system (Promega, Madison, WI, USA). PCR reactions were performed with Premix EX Taq (Takara Bio, Shiga, Japan) according to the manufacturer’s recommendations and with the following cycling conditions: initial denaturation for 30 s at 95°C, followed by 40–50 cycles of 95°C for 5 s and 60°C for 34 s, in an Applied Biosystems 7500 Real-Time PCR System (Applied Biosystems, Foster City, CA, USA). The data were analyzed using the 7500 system SDS software program (v1.4; Applied Biosystems). The following TaqMan gene expression assays were used: Hs00173908_m1 (*LGR4*), Hs00173664_m1 (*LGR5*), Hs00663887_m1 (*LGR6*), Hs00362096_m1 (*EPHB2*), Hs00270888_s1 (*ASCL2*), Hs00197437_m1 (*OLFM4*), Hs01009250_m1 (*PROM1*), Hs010780810_m1 (*CDX2*), Hs00212584_m1 (*CLDN18*), and Hs0275899_g1 (*GAPDH*). *GAPDH* served as the endogenous control. All experiments were performed in duplicate.

### Statistical analysis

Statistical analyses were performed using the PASW 18.0 statistical software program (IBM SPSS Statistics, Chicago, IL, USA) and Prism version 5.0 (GraphPad Software, Inc., San Diego, CA, USA). The correlations between *LGR5* positivity and clinicopathological parameters were tested using the χ^2^-test or Fisher’s exact test. Between-group comparisons of the real-time PCR data were performed using Student’s *t*-test. The significance of the relationship between *LGR5* and *CD133* expression was assessed with the Pearson correlation test. The correlation between the grades according to RNA ISH and the *LGR5* transcripts measured by RT-PCR was evaluated by the Spearman correlation test. The results were considered significant when *p*<0.05.

## Results

### 1. RNAscope specifically identifies *LGR5* ^+^ stem cells in human tissues

**Figure 1:**
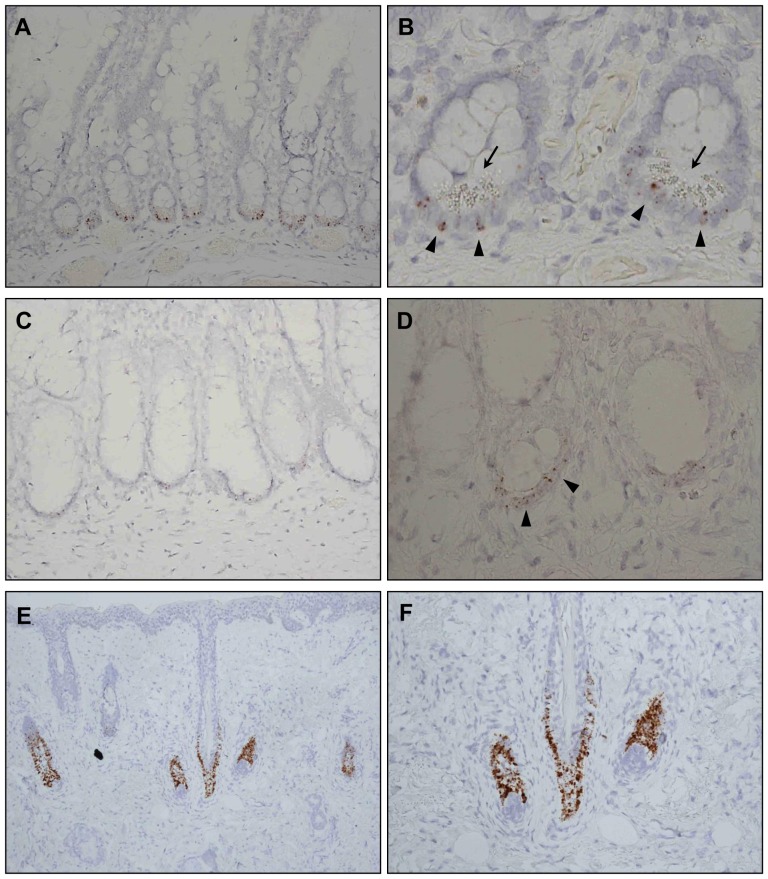
Validation of RNA *in situ* hybridization (ISH) for the identification of *LGR5* ^+^ cells in established niches in normal human tissues. RNA ISH was performed to detect *LGR5*-expressing cells in formalin-fixed and paraffin-embedded small intestine, colon, and hair follicle samples. *LGR5* ^+^ cells are indicated by the brown colored dots. (A) *LGR5* ^+^ cells were observed at the base of all small bowel crypts. (B) *LGR5* ^+^ cells, indicated by arrowheads, were located next to or between Paneth cells, which are distinguished by their characteristic cytoplasmic granules and marked by arrows. (C, D) *LGR5* ^+^ cells in the colonic crypts had lower *LGR5* expression than *LGR5* ^+^ cells in the small intestine. (E, F) Many *LGR5* ^+^ cells were noted in the hair follicle bulges. Magnifications: A, C, F, ×200; B, D, ×400; E, ×100.

*Lgr5* **^+^** cells have been well documented in the stem cell niches of knock-in mouse models, including the crypt bases of the small and large intestines and hair follicle bulges [3], [19]. However, the identification of *Lgr5* **^+^** cells in human clinical specimens has not been very successful, possibly because the sensitivities and specificities of LGR5 antibodies and RNA ISH techniques are insufficient. In the present study, we applied RNAscope, a novel RNA ISH technology, to examine human FFPE tissues [20]. To validate this method before investigating the distribution of *Lgr5* **^+^** cells in the stomach, we assessed whether RNAscope could specifically mark *Lgr5* **^+^** cells in well-established niches. As expected, groups of *Lgr5* **^+^** cells were detected at the exact intestinal and hair follicular locations that had been observed in mice. Almost every small intestinal crypt harbored several *Lgr5* **^+^** cells in the basal area (Fig. 1A), and *Lgr5* **^+^** cells were interspersed with Paneth cells (Fig. 1B). Most colon crypts contained *Lgr5* **^+^** cells, even though the *LGR5* staining intensities were weaker than in the small intestine (Fig. 1C, D). Many *Lgr5* **^+^** cells were spread along the hair follicle bulges (Fig. 1E, F). We also confirmed *LGR5* expression in tumors that arose from these tissues, such as basal cell carcinoma (Fig. S1A) and colonic adenoma (Fig. S1B), as reported in previous studies [15], [21]. These findings demonstrated the ability of RNAscope to specifically identify *Lgr5* **^+^** stem cells in human FFPE specimens. To further establish the robustness of RNAscope, we showed that CDX2-expressing tumor cells were positively stained by RNAscope while CDX2-negative adjacent normal gastric epithelial cells were not stained (Fig. S2).

### 2. IM in the stomach is associated with a marked expansion of the *LGR5* ^+^ cell population

**Figure 2:**
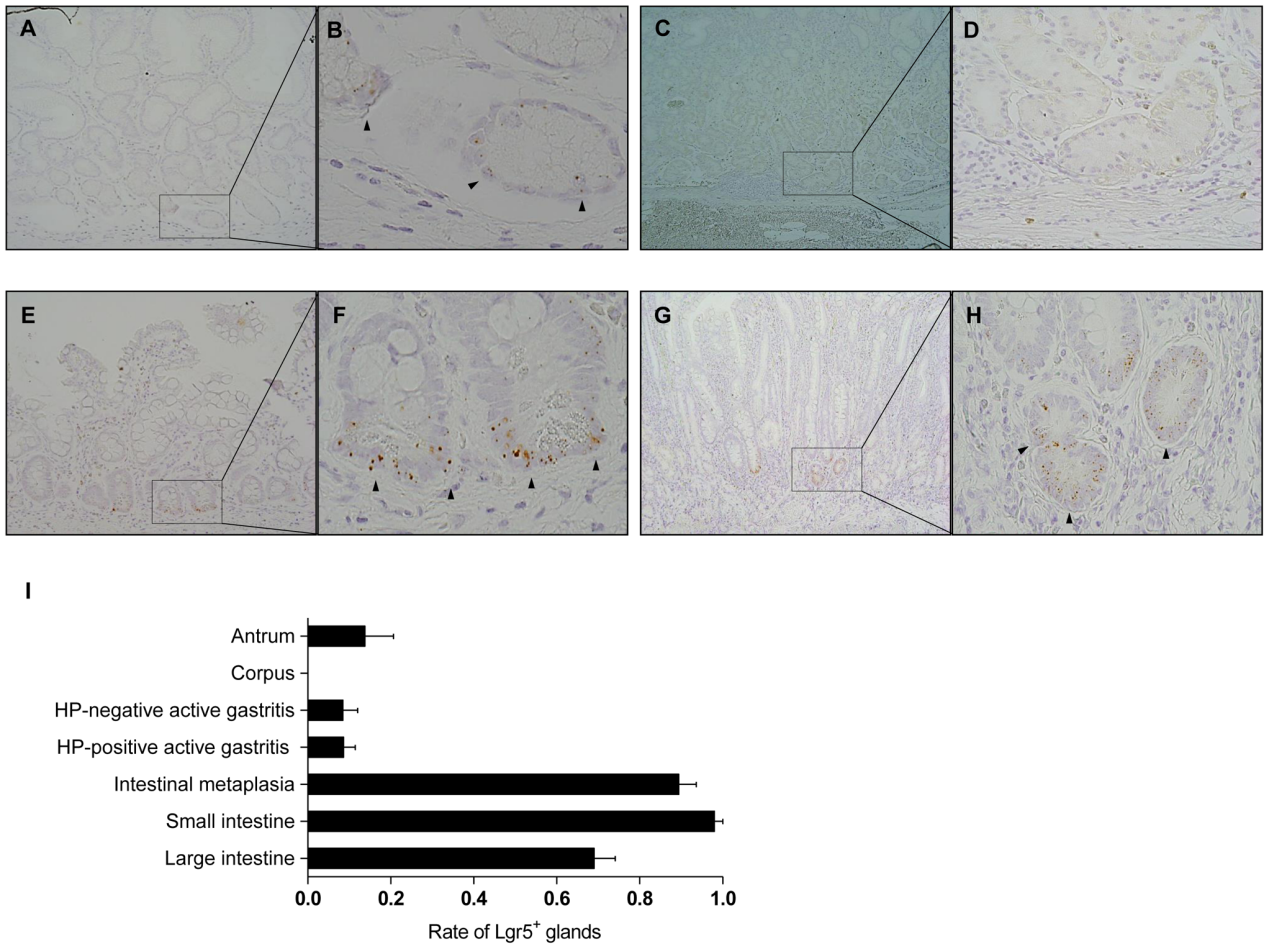
Localization of *LGR5* ^+^ cells in the gastric antrum and intestinal metaplasia. We examined many different types of non-tumorous gastric mucosa (GM), including GM without inflammation or intestinal metaplasia (IM) (antrum, n = 4; corpus, n = 4), GM with active gastritis (n = 12), and GM with IM in the antrum (n = 5), as well as small bowel (n = 5) and colon (n = 5). (A, B) Rarely, a few *LGR5* **^+^** cells were observed in the basal region of the gastric antrum. (C, D) No *LGR5* **^+^** cells were noted in the corpus. (E, F) The *LGR5* **^+^** cells population, which is located primarily in the lower regions of glands, dramatically increased in IM of the antral glands. (G, H) Interestingly, IM in the corpus had as many *LGR5* **^+^** cells as IM in the antrum. Arrows indicate the fundic glands. (E) When the ratio of *LGR5* **^+^** cell-containing glands in 20 consecutive glands was assessed, IM showed a similar ratio to the small intestine. *LGR5* **^+^** cells are indicated by arrowheads. HP, *Helicobacter pylori*. Magnifications: A, C, E, G, ×100; B, F, ×600; D, H, ×400.

We then investigated the spatial distribution of *Lgr5* **^+^** cells in the non-tumorous gastric mucosa. Consistent with the findings in mice, a sparse *Lgr5* **^+^** cell population was observed only at the base of the antral glands (Fig. 2A, B), but not at the isthmus or neck region. No *Lgr5* **^+^** cells were noted at the fundic glands (Fig. 2C, D), suggesting that *Lgr5* **^+^** cells only comprise a small group of stem cells restricted to the antrum. Remarkably, IM was associated with a dramatic increase in the number of *Lgr5* **^+^** cells (Fig. 2E, F). Interestingly, *Lgr5* **^+^** cells were also detected in IM in the corpus, where no *Lgr5* **^+^** cells were present (Fig. 2G, H). *Lgr5* **^+^** cells were mostly restricted to the basal areas of the metaplastic glands, similar to the pattern observed in the small intestine. Figure S3 shows an additional set of pictures of *Lgr5* **^+^** cells in the gastric mucosa. Active gastritis with or without *Helicobacter pylori* infection had no effect on the *Lgr5* **^+^** cell population (Fig. 2I). When counting the number of glands with *Lgr5* **^+^** cells among 20 consecutive glands in both the gastric and intestinal mucosa, we surprisingly discovered that the frequency of glands with *Lgr5* **^+^** cells in IM was almost as high as that in the small intestine (Fig. 2I), indicating that IM of the stomach seems to recapitulate intestinal mucosal features with regard to the stem cell population.

### 3. The Lgr5^+^ cell population specifically increases with IM and positively correlates with CD133 expression

**Figure 3:**
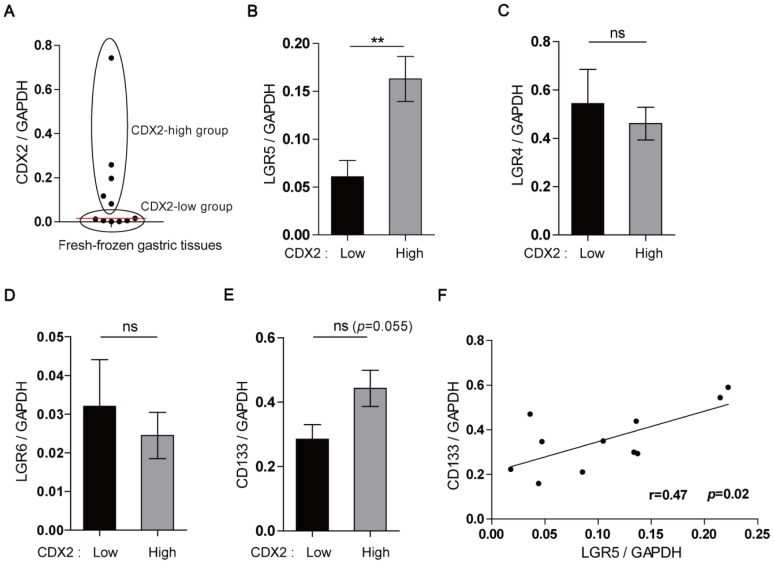
Increased *LGR5* expression with gastric intestinalization. Semi-quantitative real-time PCR was performed with fresh-frozen normal gastric tissues (n = 11) to analyze the expression of *LGR4*, *LGR5*, *LGR6*, *CDX2*, and *CD133*. (A) The samples were divided into the *CDX2*-high and -low groups according to the median *CDX2* value (red line). *LGR5* expression was significantly higher in the *CDX2*-high group (B), whereas there were no differences in the levels of *LGR4* (C) and *LGR6* (D) between the groups. (E) *CD133* expression was higher in the *CDX2*-high group, although this was not statistically significant (*p* = 0.055). (F) A positive correlation was observed between *LGR5* and *CD133* expression (Pearson correlation coefficient  =  0.47, *p* = 0.02).

To provide further evidence of the close relationship between the appearance of *Lgr5* **^+^** cells and IM, we collected 11 fresh-frozen, non-tumorous gastric tissues and divided them into 2 groups according to the *CDX2* expression level, either *CDX2*-low and *CDX2*-high, because *CDX2* is highly expressed in IM of the stomach [22] (Fig. 3A). The expression of claudin-18, known as gastric-type claudin, was also examined. However, there was no significant correlation between claudin-18 and *CDX2* expression (Fig. S4). When *LGR4, 5*, and *6* expression levels were compared between the 2 groups, only the *LGR5* level was significantly higher in the *CDX2*-high group, thus confirming a specific association between *LGR5* expression and gastric mucosal intestinalization (Fig. 3B, C, and D). The *CD133* expression level was also higher in the *CDX2*-high group, although the difference was not statistically significant (*p* = 0.055; Fig. 3E), which led us to hypothesize that the increased number of *Lgr5* **^+^** cells was associated with *CD133* expression, because *CD133* is a stem cell marker in many different types of tumors [23]. Indeed, we found a positive correlation between *LGR5* and *CD133* expression when analyzing the RT-PCR data from the normal gastric mucosa (Pearson correlation coefficient  =  0.47, *p* = 0.02) (Fig. 3F). In a mouse study, *Lgr5* **^+^** cells were the cells of origin of a Wnt-driven GA [9]. Most human GAs occur from an IM background [24], and most have *APC* gene mutations [25]. Based on these findings, we postulated that IM in the stomach enhances the risk of tumor development by increasing the *Lgr5* **^+^** cell population, which is highly susceptible to transformation by *APC* mutation.

### 4. *LGR5* ^+^ tumor cells are often confined to the basal area of tumor glands, reminiscent of normal stem cell niches

**Figure 4:**
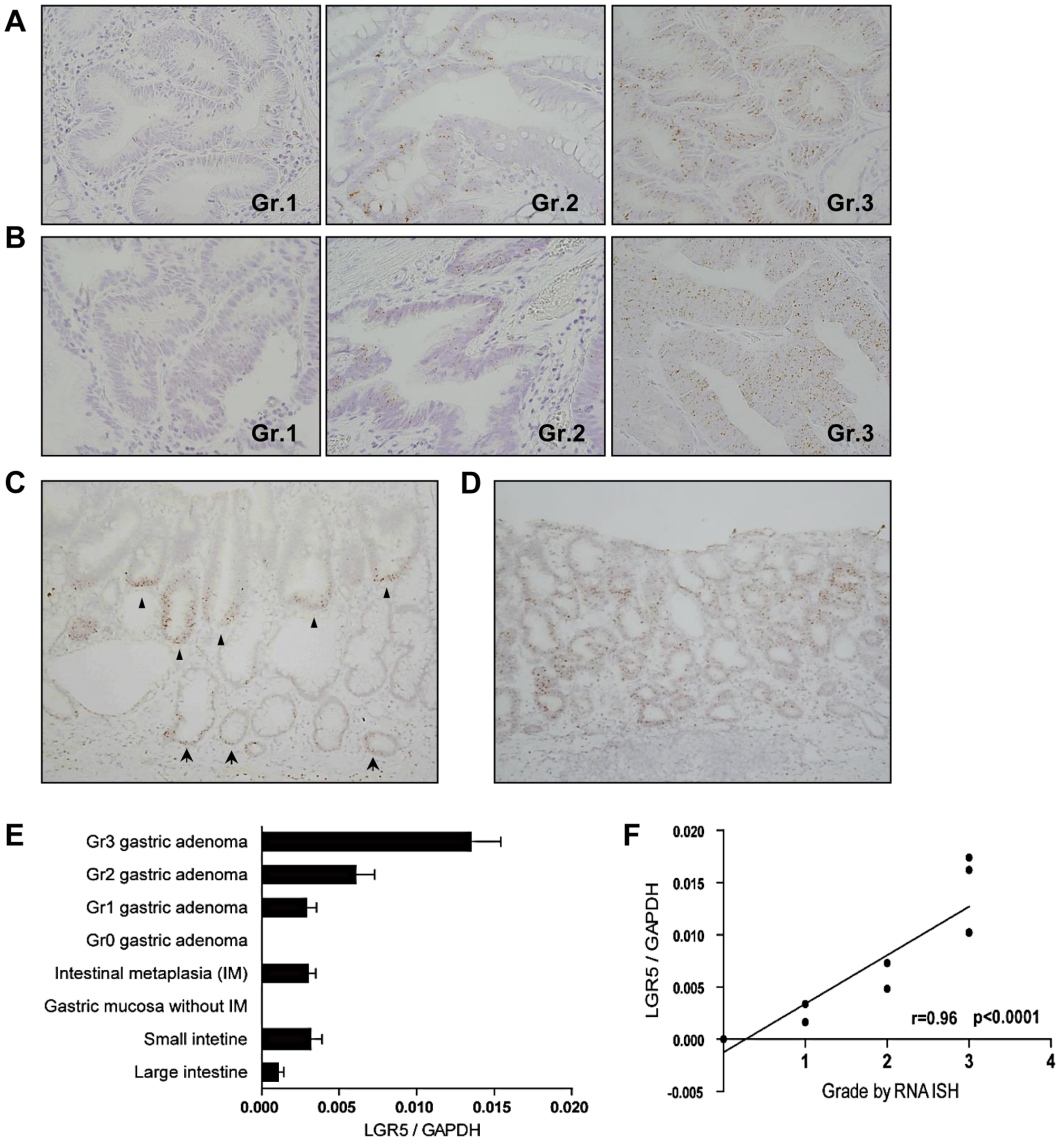
*LGR5*-expressing cells in gastric tumors. Tissue microarrays were constructed from gastric tumors, including gastric adenomas (GAs) (n = 75) and early gastric carcinomas (EAGs) (n = 68), that had been removed by endoscopic submucosal dissection. *LGR5* expression in GAs (A) and EGCs (B) was classified according to the percentage of *LGR5* ^+^ tumor cells as grades 0, 1, 2, and 3. (C) Some GAs had a distinct *LGR5* **^+^** cell distribution that was restricted to the bases of tumor glands (marked by arrowheads); this distribution was quite similar to that of *LGR5* **^+^** cells in IM (indicated by arrows) observed around the tumor. (D) However, some GAs contained *LGR5*-expressing tumor cells in a relatively diffuse or patchy distribution pattern. (E) Semi-quantitative real-time PCR from formalin-fixed and paraffin-embedded GAs (n = 11), IM in the antrum (n = 3), GM without IM in the corpus (n = 4), and small (n = 4) and large (n = 4) intestinal tissues was performed to confirm the RNA *in situ* hybridization (ISH) results. (F) There was a positive correlation between the *LGR5* grades in GAs as determined by RNA ISH and *LGR5* transcript levels in RT-PCR (Spearman correlation coefficient  =  0.96; *p*<0.0001). Magnifications: A, B, ×400.

To explore the effects of *Lgr5* **^+^** cells on tumor development in the human stomach, we constructed tissue microarrays (Fig. S5A) and examined the expression of *LGR5* in gastric tumors obtained by ESD, including GAs and early gastric carcinomas (EGCs). The *LGR5* expression levels were graded according to the percentage of *LGR5* ^+^ tumor cells (Fig. 4A, B and Fig. S5B, C). Tumors above grade 2 were considered positive. The degree and pattern of *LGR5* expression varied between the gastric tumors. The *LGR5*-positive tumors could be roughly divided into 2 types based on the distribution of *Lgr5* **^+^** cells: tumors with basal (Fig. 4C) or diffuse (Fig. 4D) patterns. In the basal pattern tumors, the *Lgr5* **^+^** cells were mostly restricted to the base of the adenoma segment, which was reminiscent of both IM and the normal crypt architecture. More than half of the tumors exhibited a basal accumulation of *Lgr5* **^+^** cells, regardless of the histological progression of the gastric tumors, although the overall frequency of *LGR5* positivity declined (Fig. S8). This observation led us to speculate that these *Lgr5* **^+^** cells function as tumor stem cells. In a mouse study, the same tendency of *Lgr5* **^+^** cells to localize toward the base of the adenoma segment was reported in the intestinal adenomas, and their stem cell-like properties were directly demonstrated by the coexpression of other stem cell markers such as *OLFM4* and *ASCL2*, as well as by stem cell activity [7]. Additionally, to confirm the results from RNA ISH, total RNAs were obtained from FFPE samples of each gastric tumor grade, non-tumorous gastric mucosa, and intestinal mucosa, and the *LGR5* transcripts were subsequently measured by semi-quantitative RT-PCR (Fig. 4E). The *LGR5* transcript levels in IM of the stomach were similar to those in the small intestine. The RNA ISH assay grades correlated well with the *LGR5* mRNA levels determined by RT-PCR (Spearman correlation coefficient  =  0.96; *p*<0.0001) (Fig. 4F).

### 5. *LGR5* positivity is associated with nuclear β-catenin, histological differentiation, and mucin type in gastric tumors

**Figure 5:**
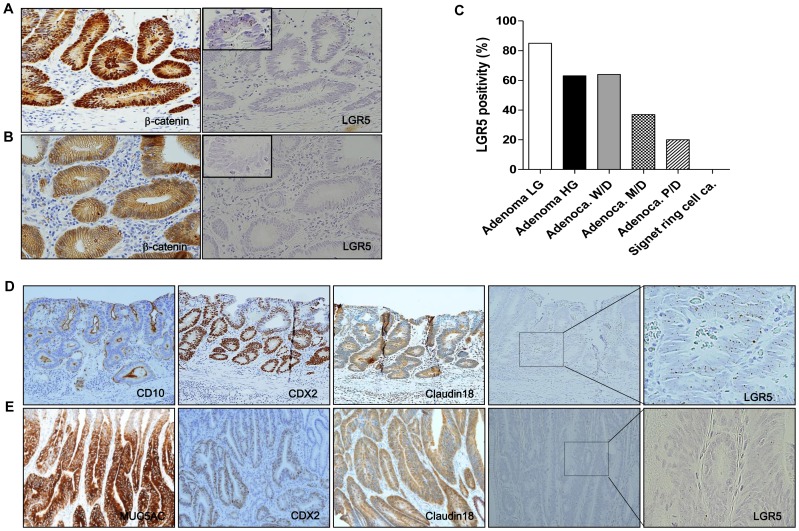
Relationships between *LGR5* positivity and nuclear β-catenin, histological differentiation, and mucinous type in gastric adenomas. Gastric adenomas with strong cytoplasmic and nuclear β-catenin expression (n = 44) were highly likely to be positive for *LGR5* (A), whereas adenomas with normal β-catenin expression levels (n = 31) had relatively low *LGR5* positivity (B). (C) As the tumors progressed and dedifferentiated, *LGR5* positivity declined (n = 143). Intestinal-type adenomas (n = 30) (A) generally had higher levels of *LGR5* expression than gastric-type adenomas (n = 13) (E). CD10 and MUC2 expression refers to the intestinal tumor gland phenotype, and MUC5AC and MUC6 mucin expression represents the gastric gland phenotype. LG, low grade; HG, high grade; Adenoca, adenocarcinoma; W/D, well differentiated; M/D, moderately differentiated; P/D, poorly differentiated. Magnifications: A, B, D, E, ×200.

Correlations of *LGR5* positivity with various clinicopathological factors were evaluated and summarized for GAs (Table 1) and EGCs (Table S1). A total of 57 GA cases (76%) and 31 EGC cases (43%) were positive for *LGR5*. *LGR5* expression was strongly and positively associated with nuclear β-catenin in both GAs (*p* = 0.015) (Fig. 5A, B and Fig. S6) and EGCs (*p* = 0.000), which was consistent with previous reports that documented a relationship between LGR5 and Wnt pathway activation [26], [27]. *LGR5* expression was higher in low-grade adenomas than in high-grade adenomas (*p* = 0.025) (Table 1), and the expression tended to decline with tumor dedifferentiation (*p* = 0.036) (Fig. 5C). Nuclear β-catenin expression also significantly decreased with tumor progression (Table S2). Moreover, *LGR5* expression was higher in adenomas with intestinal-type glands than in those with gastric-type glands (Fig. 5D and E and Fig. S7) (*p* = 0.024). Thus, our findings indicate that *Lgr5* **^+^** cells occur more frequently in low-grade tumors with active Wnt pathway signaling and an intestinal gland type, suggesting that *LGR5* is more likely involved in the very early stages of Wnt-driven tumorigenesis in the stomach.

**Table 1:** Assessment of *LGR5* expression in gastric adenomas.

	Total (%)	LGR5		p-value
		Negative (%)	Positive (%)	
**Patients**	75 (100)	18 (24)	57 (76)	
**Age**				
≥65	41 (55)	10 (24)	31 (76)	1.000†
<65	34 (45)	8 (24)	26 (76)	
**Gender**				
Female	22 (29)	4 (18)	18 (82)	0.560†
Male	53 (71)	14 (26)	39 (74)	
**Grade**				
Low	47 (63)	7 (15)	17 (85)	0.025†
High	30 (37)	11 (37)	40 (63)	
**β-catenin**				
Nuclear stain	44 (59)	6 (14)	38 (84)	0.015†
No nuclear stain	31 (41)	12 (39)	19 (61)	
**Mucinous type**				
Gastric	13 (17)	7 (54)	6 (46)	0.024^#^
Intestinal	30 (40)	4 (13)	26 (87)	
Mixed	23 (31)	5 (22)	18 (78)	
Unclassified	9 (12)	1 (11)	8 (89)	

^#^Pearson Chi-Square. ^†^Fisher's exact test.

### 6. *LGR5* expression is associated with the levels of other intestinal stem cell (ISC) markers

**Figure 6:**
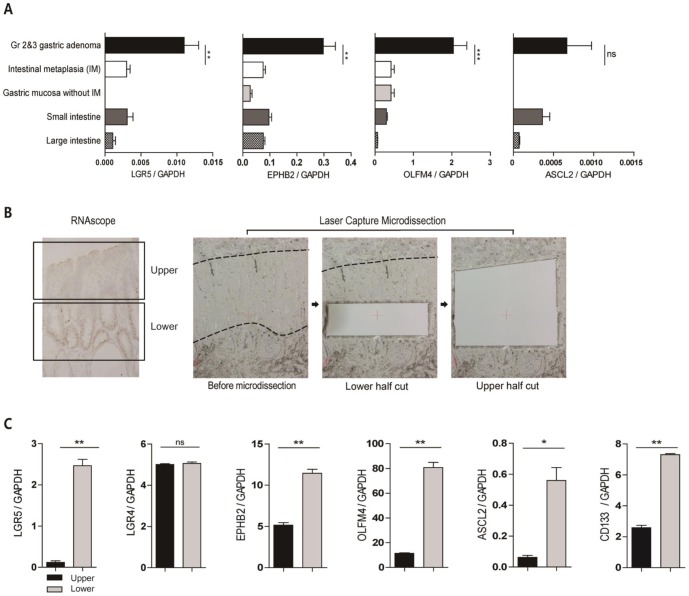
*LGR5* ^+^ cells, restricted to the lower regions of tumor glands, are associated with high levels of intestinal stem cell (ISC) markers. (A) *LGR5-*positive gastric adenomas (n = 8) expressed substantially higher levels of ISC markers such as *EPHB2*, *OLFM4,* and *ASCL2* than non-tumorous gastric mucosa (IM, n = 4; GM without IM in the corpus, n = 4) and the small (n = 3) and large (n = 3) intestines, although the difference in *ASCL2* expression was not statistically significant. (B) Lower and upper regions were laser-capture microdissected from GAs with basal *LGR5* ^+^ cells. (C) Semi-quantitative real-time PCR of the dissected tissues revealed that the basal regions that harbored the most *LGR5* ^+^ cells had higher levels of ISC markers and *CD133*, whereas no difference in *LGR4* expression was observed between the lower and upper regions.

The distinct basal restriction of *LGR*5**^+^** cells in some GAs is interesting because it strikingly resembles the restriction of *Lgr5* **^+^** stem cells to the niche in normal tissues. To further investigate whether the *Lgr5* **^+^** cells in human GAs had any stem cell properties, we analyzed the levels of ISC markers such as *ASCL2*, *EPHB2*, and *OLFM4*, which are highly expressed in stem-like cells from human colorectal cancers [28], as well as in murine intestinal adenomas [7]. In general, GAs expressed significantly higher levels of *EPHB2* and *OLFM4* than the non-tumorous gastric mucosa (Fig. 6A). To clarify whether the upregulated expression of ISC markers was related to the *LGR5* ^+^ tumor cells, we microdissected the tumor glands into upper and lower regions (Fig. 6B) and compared the marker transcript levels in both areas. The results confirmed that *LGR5* expression was higher in the lower region, whereas the expression of *LGR4*, which is a close relative of the *LGR5* gene, did not differ between the 2 regions (Fig. 6C). The expression levels of all examined ISC markers were higher in the lower region, where the majority of *Lgr5* **^+^** cells exist (Fig. 6C). Furthermore, *CD133* expression was higher in the basal regions of the tumor glands (Fig. 6C). Thus, these findings support the hypothesis that *Lgr5* **^+^** cells restricted to the bases of tumor glands retain stem cell properties in human GAs.

### 7. Basal restriction of *LGR5* ^+^ cells in GAs correlates with the differential expression of ISC markers along the gland axes

**Figure 7:**
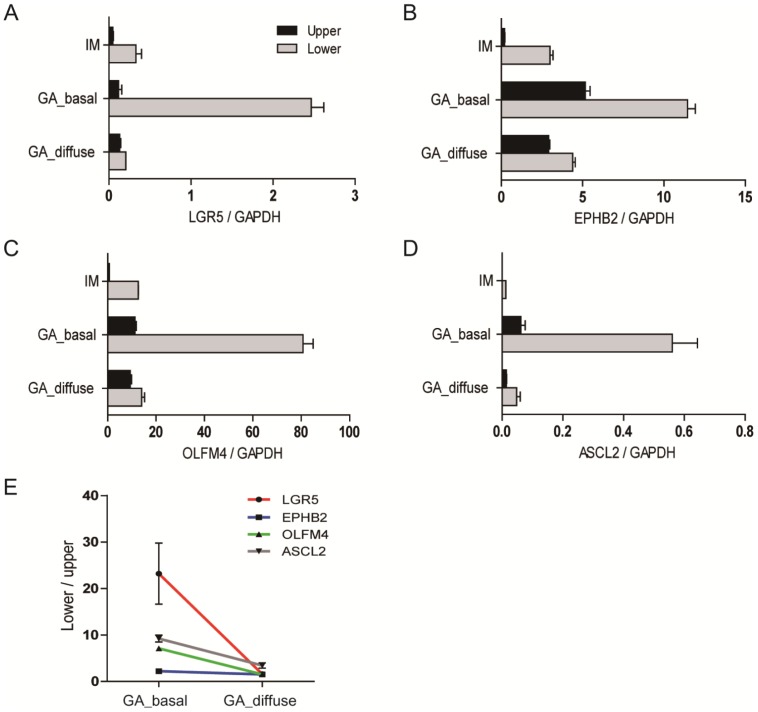
Comparison of ISC marker expression in gastric adenomas with different *LGR5* distribution patterns. The upper and lower gland regions were selectively microdissected from IM (n = 3) and the GA with diffusely distributed *LGR5* **^+^** cells (GA_diffuse) to compare the differential expression of ISC markers to those of the GA with basally restricted *LGR5* **^+^** cells (GA_basal). RT-PCR analysis of *LGR5* (A), *EPHB2* (B), *OLFM4* (C), and *ASCL2* (D) expression demonstrated that the ISC marker expression gradient between the lower and upper gland regions was remarkably reduced in the GA with a diffuse pattern (E).

To strengthen the hypothesis that the *Lgr5* **^+^** cells restricted to the bases of tumor glands are essential for the distinct ISC marker expression patterns in the upper and lower regions, we examined a GA with relatively diffuse *LGR5* distribution and a slight basal accentuation of *LGR5* expression. *LGR5* expression in the upper and lower halves of the gland differed little in the adenoma, unlike that in IM and GAs with basal *LGR5* expression (Fig. 7A). Indeed, the wide regional variation in the expression of all ISC markers that was observed in the GAs and IM with basal *LGR5* expression was remarkably reduced in the GA with diffuse *LGR5* expression (Fig. 7B, C, and D). The expression ratios of the lower regions to the upper regions for all ISC markers, including EphB2 (*p* = 0.018), *OLFM4* (*p* = 0.002), and *ASCL2* (*p* = 0.025), were significantly decreased in the GA with diffuse *LGR5* expression, compared to a GA with basal *LGR5* expression (Fig. 7E). These findings indicate that the basal restriction of *Lgr5* **^+^** cells in GAs is closely correlated with the gradient expression of ISC markers along the gland axes.

### 8. Spatial correlation of LGR5 expression with ISC markers in GAs

**Figure 8:**
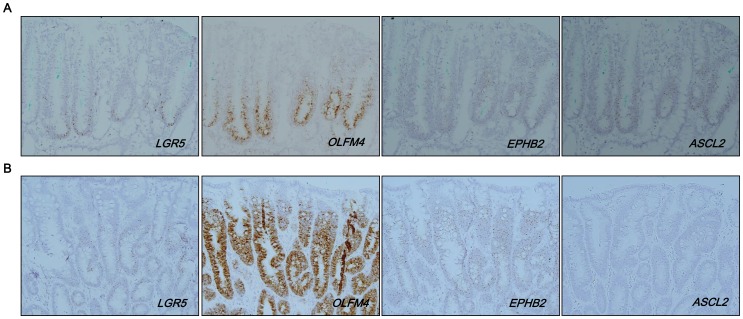
Correlation of *LGR5* ^+^ cells with other intestinal stem cell markers. In RNA *in situ* hybridization, intestinal stem cell markers (*OLFM4, EPHB2*, and *ASCL2*) were mainly expressed at the basal area of tumor glands in the GA with a basal pattern (A). In contrast, the GA with a diffuse pattern (n = 2) showed diffuse expression of the markers throughout the tumor (B). Magnification: A, B, ×200.

To confirm the RT-PCR results that indicated an association between *LGR5* expression and the expression of other ISC markers, we performed RNA *in situ* hybridization on GAs with basal or diffuse patterns. For a GA with a diffuse pattern, we selected one with strong and diffuse *LGR5* expression for improved visualization at a low power view. The expression of *OLFM4*, *EPHB2*, and *ASCL2* was relatively restricted to the basal region of tumor glands in the GA with basal *LGR5* expression (Fig.8A), but the expression of these markers was diffuse in the GA with a diffuse *LGR5* expression pattern (Fig. 8B). Notably, in the GA with a basal pattern, *LGR5* expression was confined to the very lower area of the tumor glands when other stem cell markers were expressed by a larger population of cells beyond the base (Fig. 8A and Fig. S9), suggesting that *LGR5* is a marker for a specific group of cells with stem cell features. On the other hand, for the GA with diffuse LGR5 expression in which the majority of tumor cells expressed ISC markers as well, it remains unclear whether all *Lgr5* **^+^** cells are tumor stem cells.

## Discussion

Considerable evidence supports that *Lgr5* is a normal gastrointestinal tract stem cell marker in mice, with a corresponding role in murine tumors. Investigations of *LGR5* expression in human tissues, however, have been limited, and an appropriate histological method to identify *Lgr5* **^+^** cells in human organs has not been established. Previous studies that attempted to use antibodies or RNA ISH on human FFPE tissues did not seem to provide accurate results with regard to the specific labeling of LGR5**^+^** cells [10]–[15]. For example, Uehara et al. [29] and Wu et al. [30] recently reported the distribution of LGR5 cells in human gastric glands using commercially available LGR5 antibodies. However, an appropriate positive control stain to validate the specificity of the antibodies was not provided, and the results showed nonspecific staining of parietal cells or hematopoietic cells in the lamina propria of the stomach. Simon et al. generated monoclonal antibodies to LGR5 to investigate the histo-anatomical distribution of Lgr5^+^ cells with gastric cancer progression and validated its specificity by western blot analysis of LGR5-transfected cell lines [11]. However, the immunostaining of colonic mucosa as a positive control was not entirely convincing because of the strong positive staining of stromal and endothelial cells. More importantly, none of these studies demonstrated the specific localization of LGR5 cells at the basal glands of normal gastric antrum, as shown in mice. Nakata et al. [31] and Becker et al. [12] did provide a positive control stain for LGR5 antibodies with the intestinal mucosa before using the antibodies in brain and colonic tumors, but the quality of the staining was not sufficient to ensure the sensitivity and specificity of the antibodies. Although RNA ISH has specifically shown the *Lgr5* **^+^** cells restricted at the crypt base in studies of human and murine intestines, the visualization quality of Lgr5^+^ cells in FFPE tissues was not satisfying, and the technique has not been used in human gastric tissues [14], [15], [32].

In the present study, we used RNAscope to determine the presence of *Lgr5* **^+^** cells in various gastric lesions after validating the technique. We specifically identified *Lgr5* **^+^** cells at the same locations in human gastric mucosa, intestines, and hair follicles as previously shown in mice. Moreover, the quality of the staining was much better than with conventional RNA ISH, which made it easier to evaluate *Lgr5* **^+^** cells in their cellular context. Consequently, we expect that this method will facilitate studies of *Lgr5* **^+^** cells in other archived human samples, which could in turn accelerate investigations into the practical significance of LGR5 in a variety of human diseases. However, RNA ISH only detects cells that contain *LGR5* transcripts; it is unknown whether *LGR5* transcripts are sufficiently translated into proteins that play functional roles in determining stem cell properties. It remains a possibility that *LGR5* serves mainly as a surrogate stem cell marker without any functional implications. Although LGR5 acts as a receptor for ligands such as R-spondins and helps augment Wnt signaling [33], [34], the functional relevance of LGR5 in humans with regard to stem cell activity has yet to be determined.

We observed *Lgr5* **^+^** cells at almost all crypts of the small intestine and colon and at the hair follicle bulges, which confirmed that *Lgr5* **^+^** cells are a major stem cell population in these tissues. However, *Lgr5* **^+^** cells were barely detectable in the antral glands of the normal gastric mucosa, suggesting that *Lgr5* **^+^** cells in the stomach comprise only a small fraction of the total stem cell population. The stomach can be divided into 4 major parts, depending on its histo-anatomical features, and all proposed stem cell markers to date are present in a limited area of the stomach [35]. Therefore, the stomach likely contains many heterogeneous stem cell populations with distinct characteristics and different markers. The intestinal epithelium is also believed to contain 2 distinct pools of stem cells, the *Bmi1*-expressing +4 cells and the *Lgr5* **^+^** crypt-based columnar cells [36].

*Lgr5* **^+^** cells in the normal antral glands were sparse and unaffected by inflammation or *Helicobacter pylori* infection. In contrast, the number of *Lgr5* **^+^** cells and the staining intensity were remarkably increased in IM to nearly the same levels observed in the small intestine. Interestingly, *Lgr5* **^+^** cells also appeared in IM of the fundic glands, in which *Lgr5* **^+^** cells are normally absent, implying that intestinalized gastric epithelial cells, including the *Lgr5* **^+^** cells in IM, might not arise from the existing *Lgr5* **^+^** stem cells. Spasmolytic polypeptide-expressing metaplasia (SPEM), another type of stomach metaplasia, does not arise from Lgr5**^+^** cells either [37]. Instead, SPEM transdifferentiates from mature chief cells in the murine [38] and human [39], [40] stomach. Moreover, Lgr5**^+^** stem cells arise from *Bmi-1*-expressing stem cells in the intestinal epithelium [36]. Collectively, these findings suggest that the *Lgr5* **^+^** cell expansion in IM arises from another group of stem cells, although no stem cell population is yet known to generate *Lgr5* **^+^** cells in the stomach.

Intestinalized gastric glands are similar in many aspects to the small intestinal epithelium. However, IM is not identical to the intestinal mucosa. One significant difference is that Paneth cells are less frequently found in IM. Paneth cells may be essential for the maintenance of *Lgr5* **^+^** stem cells in intestinal crypts [41]. However, in IM, *Lgr5* **^+^** cells usually exist in the absence of Paneth cells, indicating that *Lgr5* **^+^** cells in IM do not rely on Paneth cells as a source of signaling factors, such as Wnt ligands, Notch ligands, and epidermal growth factor, for survival. Even when Paneth cells are present in IM and GAs, *Lgr5* **^+^** cells are not necessarily located next to Paneth cells (Fig. S10). Instead of Paneth cells, *Lgr5* **^+^** cells have redundant sources of survival signals. For instance, Wnt ligands from mesenchymal cells can compensate for the loss of signals from Paneth cells [42], and c-kit^+^ secretory cells have been identified as a colonic counterpart of Paneth cells that could support *Lgr5* **^+^** stem cells [43]. Additionally, it was recently shown that Paneth cells are not required to sustain *Lgr5* **^+^** cells *in vivo* [44]. Thus, *Lgr5* **^+^** stem cells in IM might be maintained by an undefined epithelial or mesenchymal cell population that substitutes for Paneth cells.

We speculate that the marked increase in the number of *Lgr5* **^+^** cells during the process of IM has profound biological significance for tumor initiation in the stomach. A study of *Cdx2*-transgenic mice demonstrated that IM plays a significant role in the genesis of gastric carcinoma and that the cancer cells originated from intestinal metaplastic epithelial cells that had been entirely transformed from gastric cells by Cdx2 [45]. In addition, an increased number of *Lgr5* **^+^** cells were observed in the normal intestinal tissues of *Apc* mutant mice and proposed as the cause of more severe polyposis [14]. Accordingly, given that *Lgr5* **^+^** cells are the cells of origin of Wnt-driven tumors in the murine stomach [9], the expanded pool of *Lgr5* **^+^** stem cells might be one of the major factors of IM that contributes to tumor development in the human stomach. However, because a lineage tracing study is not applicable for human tissues at this time, direct evidence that human GAs derive from *Lgr5* **^+^** cells cannot be obtained. Therefore, future studies on the relationship of *Lgr5* **^+^** cells and GA development are required.

*LGR5* ^+^ cells were present in human GAs, where they accounted for 0 to >90% of the tumor cells with 2 main distribution patterns. In addition to tumors with a basally restricted pattern of *LGR5* ^+^ cells, we also observed adenomas with *Lgr5^+^* cells that were dispersed throughout the tumor mass; this was not noted in a mouse study, in which approximately 5–10% of the tumor cells in most of the intestinal adenomas were *Lgr5* **^+^** cells, which were mainly located at the basal areas of the tumor glands [7]. This was probably because the intestinal adenomas in the mice all arose from the same *Apc* mutation, whereas human adenomas develop in response to many different types of Wnt-pathway abnormalities, likely in combination with other genetic abnormalities, which results in tumors with different levels of Wnt signaling and varying numbers of *Lgr5* **^+^** cells. In fact, the level of *Lgr5* expression in adenomas of 2 intestinal tumorigenesis mouse models, *Apc* ^1322T^ (1322T) and *Apc* ^R850X^ (Min), differed: 1322T tumors had higher *Lgr5* expression levels than Min tumors, and more than half of the epithelial portion of the adenomas showed *Lgr5* expression [14]. Therefore, in GAs, both the distribution pattern and the number of *Lgr5* **^+^** cells are likely to differ according to the degree of Wnt pathway activation.

In most of the low-grade adenomas, at least 5% of the tumor cells were *LGR5* ^+^ cells. We might consider *Lgr5* **^+^** cells in the niches of normal tissues as stem cells; however, *Lgr5* **^+^** cells within a tumor mass do not necessarily serve as tumor stem cells. EPHB2- and LGR5-enriched cells reportedly comprise a stem-like cell population in human colorectal cancers [28]. However, it has not been evaluated whether *Lgr5* **^+^** cells in human gastric tumors retain stem cell properties. To investigate whether *Lgr5* **^+^** cells in gastric tumors had any stem cell features, we selected GAs with basally restricted *Lgr5* **^+^** cells that were reminiscent of the normal crypt and IM, and then divided the tumor glands into upper and lower regions by laser capture microdissection. Our study demonstrated that the basal regions of tumor glands, which have a majority of the *Lgr5* **^+^** cells, express significantly higher levels of ISC markers and *CD133* than the upper region. In contrast, the differential expression of ISC markers was substantially attenuated in GAs with diffusely distributed *Lgr5* **^+^** cells, which was confirmed by the co-localization of *LGR5* and ISC markers in RNA *in situ* hybridization. These findings indicate that *LGR5* expression is closely linked to increased expression of ISC markers and provide compelling evidence that basally restricted *Lgr5* **^+^** cells in GAs act as stem cells. However, the question remains whether the *Lgr5* **^+^** cells that are distributed diffusely across the adenoma and co-express ISC markers also have stem cell features; they seem too numerous to be stem cells and lack the spatial restriction to the base of the glands. If the diffuse expression only derives from strong Wnt pathway activation, as we mentioned earlier, *LGR5* may not be appropriate for use as a stem cell marker. Clearly, further studies are required to unravel the stem cell properties of *Lgr5* **^+^** cells in gastric tumors.

Although we have successfully demonstrated the stem cell phenotype of *Lgr5* **^+^** cells in GAs, there are certain limitations regarding the interpretation and generalization of our findings. First, because technical limitations precluded the specific microdissection of *Lgr5* **^+^** cells, we compared the levels of ISC markers in the upper and lower regions of tumor glands and not in *LGR5*-positive and -negative tumor cells. Thus, we cannot exclude the possibility that other stem cell populations are present and intermingled with *Lgr5* **^+^** cells in the lower regions of the gland; these cells might affect the results of stem cell-related gene expression profiling. Second, we only analyzed low-grade GAs with basally restricted *Lgr5* **^+^** cells, which represent a very early stage during intestinal-type gastric carcinogenesis. Thus, it remains unknown whether *Lgr5* **^+^** cells retain their stem cell characteristics during tumor progression. The finding that *LGR5* positivity declines with tumor progression and dedifferentiation suggests that *Lgr5* **^+^** cells no longer act as tumor stem cells during the later stages of tumor progression. In fact, tumor stem cells might evolve during tumor progression in response to changing environmental cues [46]. Finally, functional experiments were not performed in this study; instead, indirect phenotypic features related to stem cell-related gene expression patterns were investigated. Nevertheless, we believe our findings provide valuable data that support the use of *LGR5* as a stem cell marker.

## Supporting Information

Figure S1 ***LGR5*** **^+^ cells in the basal cell carcinoma of skin and adenoma of colon.** (A) *LGR5* stem cells (marked by arrow) are seen at the bulge of hair follicle, and the majority of tumor cells of basal cell carcinoma (indicated by arrow heads) which developed nearby express *LGR5*. (B) Colonic adenoma cells exhibit *LGR5* expression. Inlet pictures show a representative area at higher magnification. Magnification: A, ×100; B, ×200.
(TIF)

Figure S2 **Validation of RNAscope with** ***CDX2*** **expressing gastric adenomas.** *CDX2* expressing gastric adenoma cells (indicated by arrows), shown as strong nuclear stain by immunohistochemistry (A and C), are specifically identified by RNAscope (B and D), whereas normal gastric epithelial cells adjacent to tumor cells (indicated by arrow heads), negative for *CDX2*, do not express *CDX2* transcripts. Original magnification: A, B, C, D ×200.
(TIF)

Figure S3 **Expanded population of** ***LGR5*** **^+^ cells in the intestinal metaplasia of the stomach** (A) A few *LGR5* **^+^** cells are located at the basal area of antral glands. (B) No *LGR5* **^+^** cells are found in the body. (C) The *LGR5* **^+^** cell population remarkably increases when IM occurs in gastric antrum. (D) Notably, *LGR5* **^+^** cells also appear at the basal part of metaplastic glands that have developed in the body. Arrows indicate the fundic glands. *LGR5* **^+^** cells are indicated by arrowheads. Magnifications: A, B, C, D, ×400.
(TIF)

Figure S4 ***Claudin-18*** **expression in non-tumorous gastric mucosa.** (A) RT-PCR shows a differential expression of *CLD18* in fresh-frozen gastric tissues. (n = 11) (B) *CDX2* low group tends to express higher *CLD18* than *CDX2* high group although it is not statistically significant (*p* = 0.399).
(TIF)

Figure S5 ***LGR5*** **expressing cells in gastric tumors.** (A) Tissue microarrays were constructed from gastric tumors. *LGR5* expression in GAs (B) and EGCs (C) was classified according to the percentage of *LGR5* ^+^ tumor cells as grades 0, 1, 2, and 3. Magnification: A, B, C, D, ×200.
(TIF)

Figure S6 **Relationship between** ***LGR5*** **expression and nuclear β-catenin** Gastric adenomas with strong cytoplasmic and nuclear β-catenin expression tend to be positive for *LGR5* (A), whereas adenomas with normal β-catenin levels show relatively low *LGR5* positivity (B). Inlet pictures show a representative area at higher magnification. Magnifications: A, B, ×200.
(TIF)

Figure S7 **Correlation between** ***LGR5*** **positivity and mucinous type of gastric adenomas.** Intestinal-type adenomas (A) have higher levels of *LGR5* expression than gastric-type adenomas (B). CD10, CDX2 and MUC2 expression refers to the intestinal tumor gland phenotype and MUC5AC mucin expression represents the gastric gland phenotype. Magnifications: A, B, ×200.
(TIF)

Figure S8 **Basal arrangement of** ***LGR5*** **^+^ cells in gastric tumors.** *LGR5* expressing tumor cells are often restricted at the basal part of tumor glands or at the interface between muscularis mucosa and submucosa in the gastric tumors including low grade adenoma (A), high grade adenoma (B), well differentiated adenocarcinoma (C), and moderately differentiated adenocarcinoma (D). (E) Around half of tumors showed basal distribution pattern of *LGR5* ^+^ cells regardless of histological type. Magnification: A, B, D ×200; C ×100.
(TIF)

Figure S9 **Spatial correlation of** ***LGR5*** **expression with intestinal stem cell markers in a gastric adenoma with basally distributed** ***LGR5*** **^+^ cells**. *OLFM4*, *EPHB2*, and *ASCL2* expressions tend to gradually increase along the axis of tumor glands in a gastric adenoma in which *LGR5* ^+^ cells are restricted at the base of tumor. Magnification: A, B, C, D, ×100.
(TIF)

Figure S10 **Spatial relationship of** ***LGR5*** **^+^ cells with regard to Paneth cells.** To analyze the association of *LGR5* ^+^ cells with Paneth cells, we performed combined RNA ISH and IHC. *LGR5* ^+^ cells are marked with brown dots and Paneth cells are indicated by α-defensin stain as red in the cytoplasm. (A, B) In the small intestine, *LGR5* ^+^ cells are always adjacent to Paneth cells at the bas β e of crypts. (C, D) However, some *LGR5* ^+^ cells that appear in the IM are not located near Paneth cells. (E, F) *LGR5* ^+^ gastric adenoma cells tend to locate in the tumor regardless of adenoma Paneth cells. Boxed areas in figure A, C, and E are shown at higher magnification in figure B, D, and F respectively. *LGR5* ^+^ cells are marked by arrow heads. Magnification: A, C, E ×100; B, D, F ×400.
(TIF)

Table S1 **Assessment of** ***LGR5*** **expression in early gastric carcinomas.**
(TIF)

Table S2 **LGR5 and nuclear** β**-catenin with the progression of gastric cancers.**
(TIF)
