# Supplementary figures and images for: Distribution of LGR5 + Cells and Associated Implications during the Early Stage of Gastric Tumorigenesis
Source: PLoS One. 2013 Dec 10;8(12):e82390. doi: 10.1371/journal.pone.0082390 (PMC3858308; doi:10.1371/journal.pone.0082390)

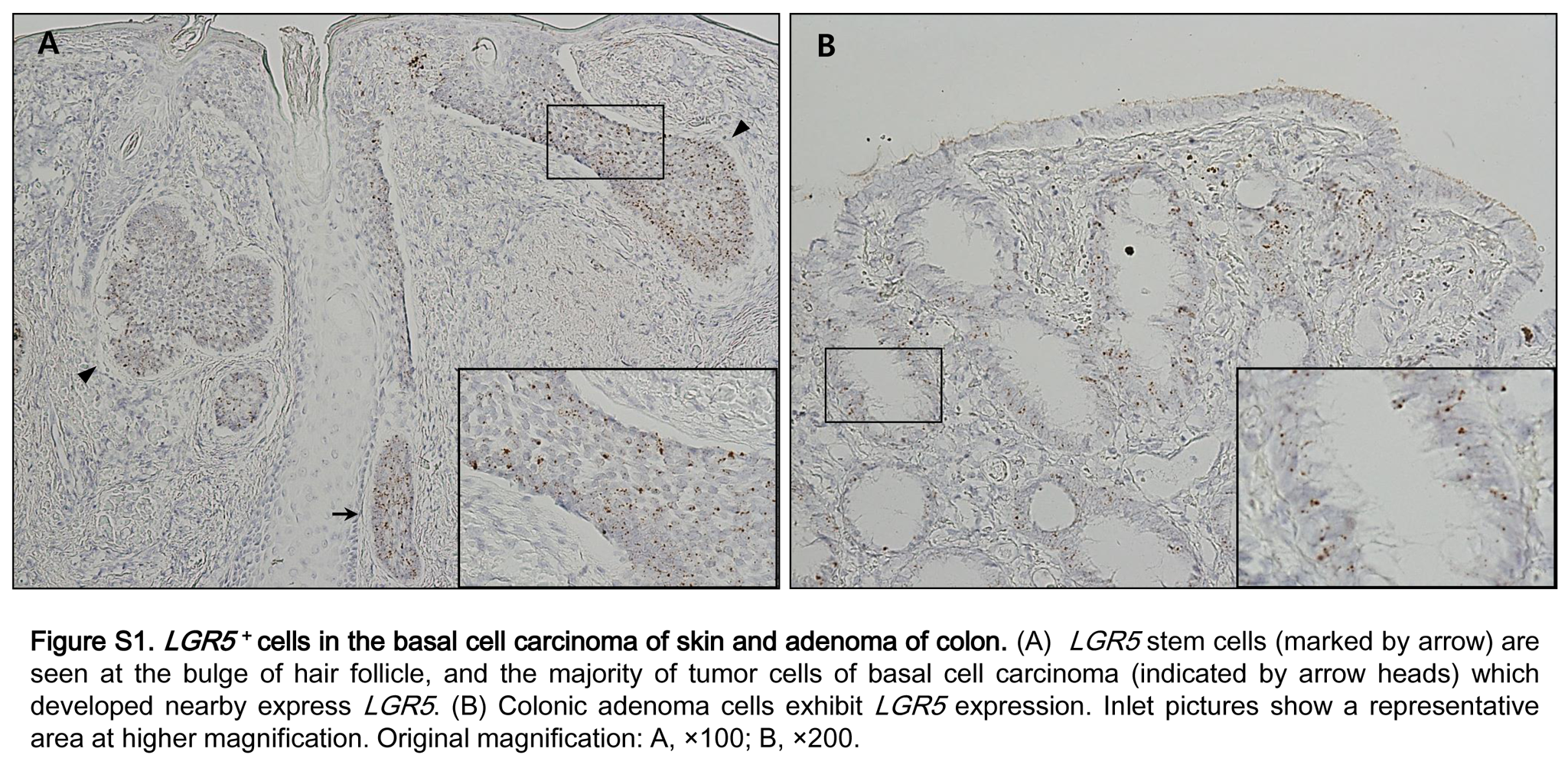

Supplement: Figure S1 — LGR5 + cells in the basal cell carcinoma of skin and adenoma of colon. (A) LGR5 stem cells (marked by arrow) are seen at the bulge of hair follicle, and the majority of tumor cells of basal cell carcinoma (indicated by arrow heads) which developed nearby express LGR5. (B) Colonic adenoma cells exhibit LGR5 expression. Inlet pictures show a representative area at higher magnification. Magnification: A, ×100; B, ×200. (TIF) [file pone.0082390.s001.tif]

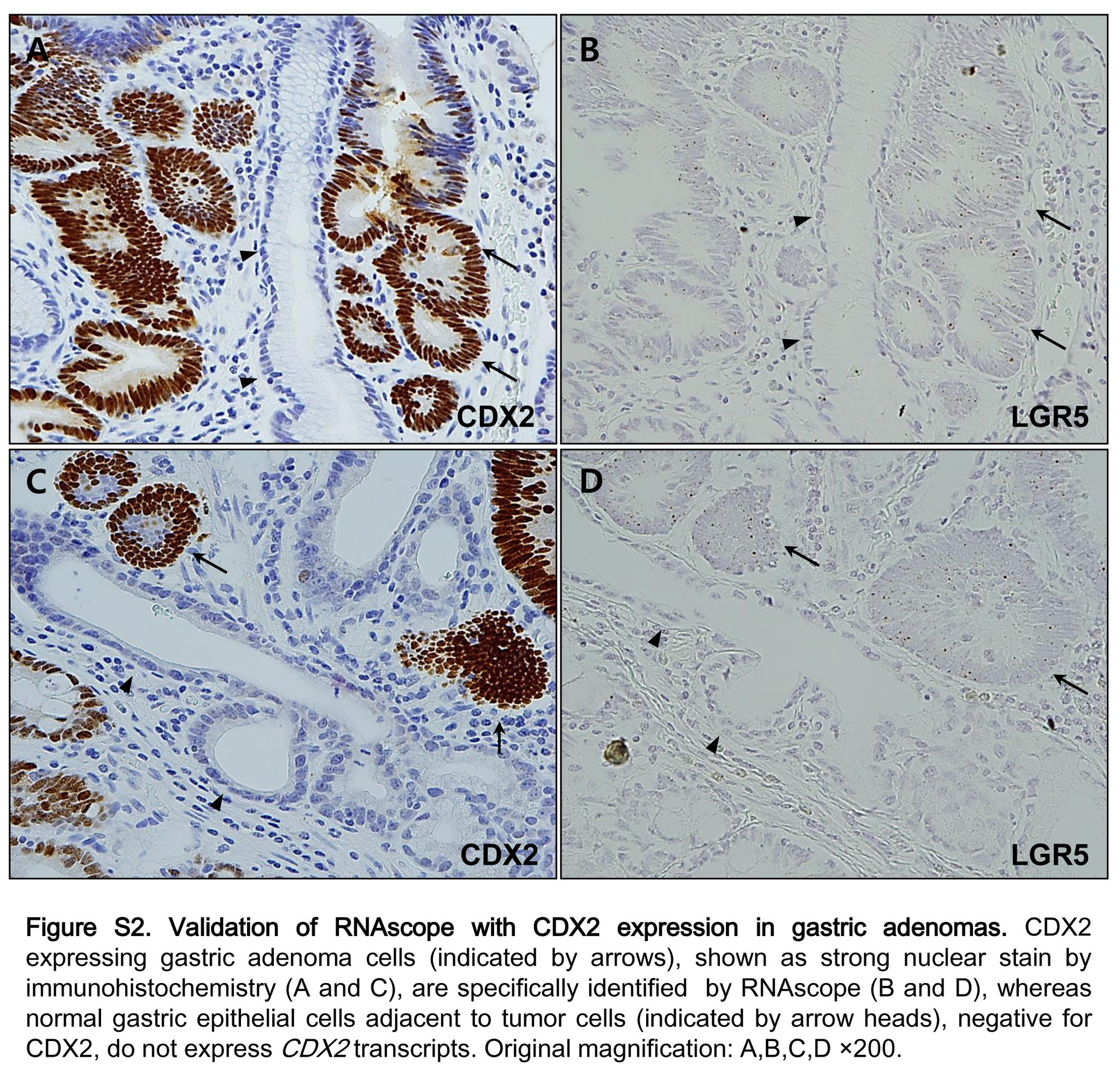

Supplement: Figure S2 — Validation of RNAscope with CDX2 expressing gastric adenomas. CDX2 expressing gastric adenoma cells (indicated by arrows), shown as strong nuclear stain by immunohistochemistry (A and C), are specifically identified by RNAscope (B and D), whereas normal gastric epithelial cells adjacent to tumor cells (indicated by arrow heads), negative for CDX2, do not express CDX2 transcripts. Original magnification: A, B, C, D ×200. (TIF) [file pone.0082390.s002.tif]

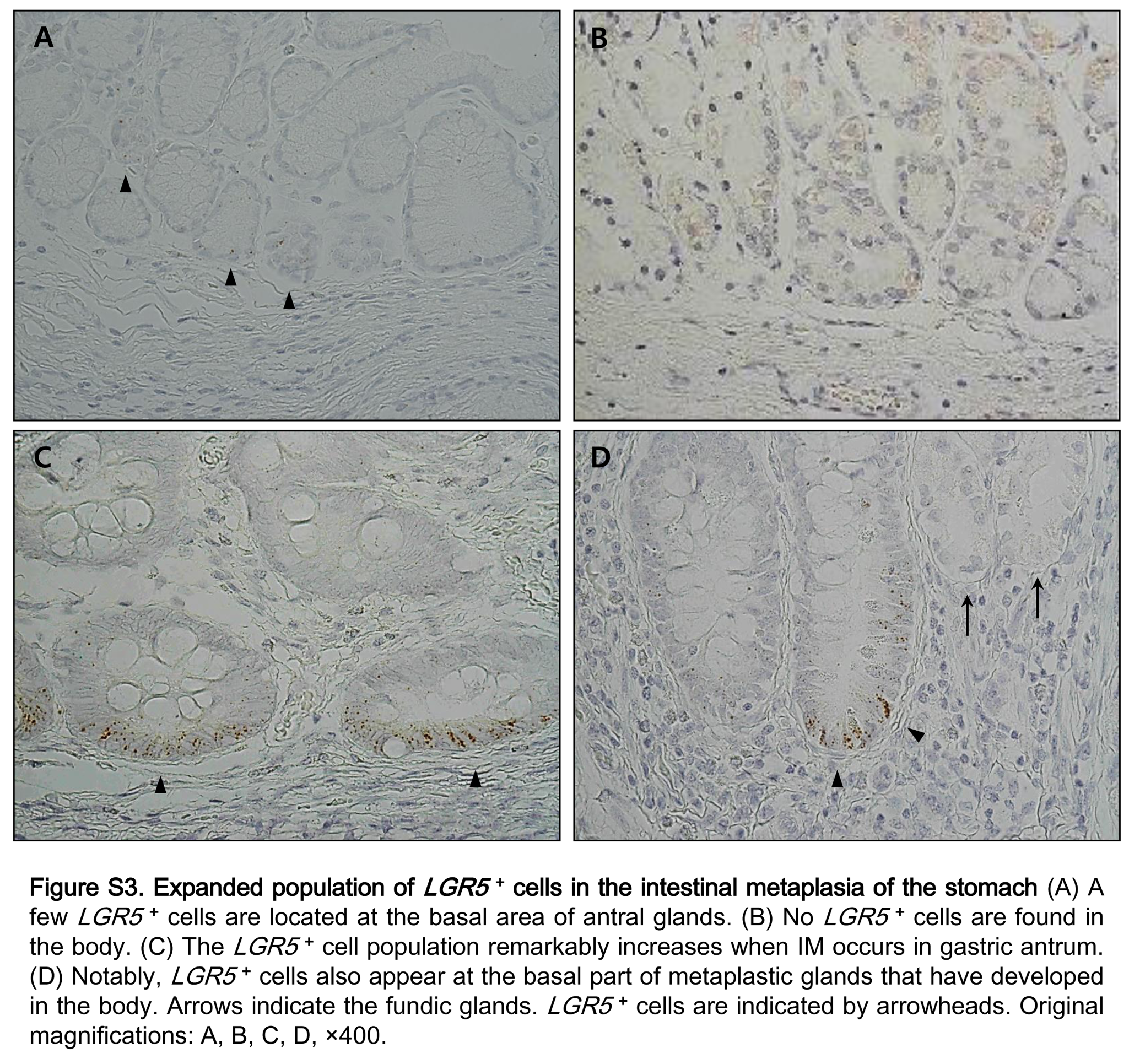

Supplement: Figure S3 — Expanded population of LGR5 + cells in the intestinal metaplasia of the stomach (A) A few LGR5 + cells are located at the basal area of antral glands. (B) No LGR5 + cells are found in the body. (C) The LGR5 + cell population remarkably increases when IM occurs in gastric antrum. (D) Notably, LGR5 + cells also appear at the basal part of metaplastic glands that have developed in the body. Arrows indicate the fundic glands. LGR5 + cells are indicated by arrowheads. Magnifications: A, B, C, D, ×400. (TIF) [file pone.0082390.s003.tif]

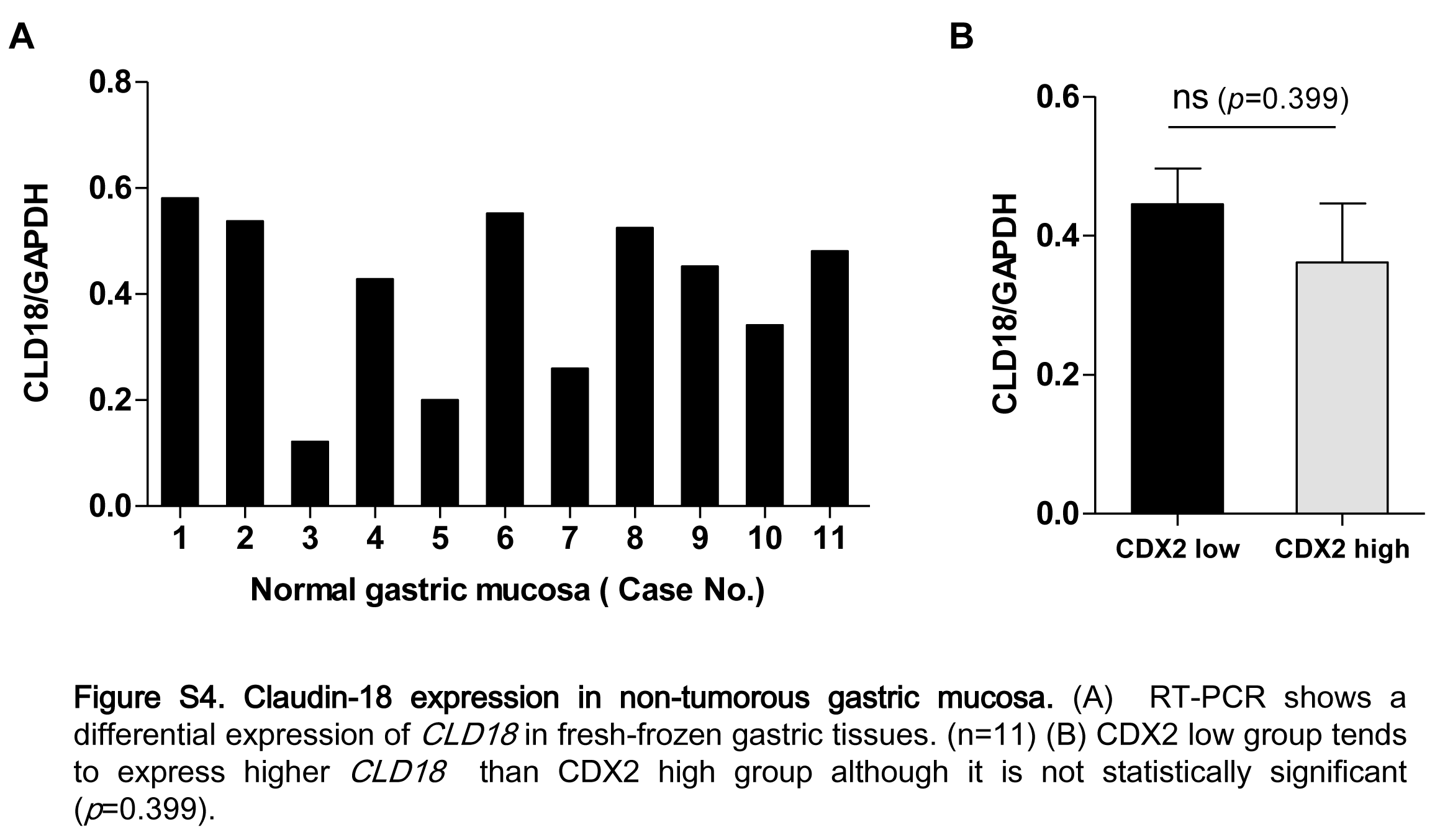

Supplement: Figure S4 — Claudin-18 expression in non-tumorous gastric mucosa. (A) RT-PCR shows a differential expression of CLD18 in fresh-frozen gastric tissues. (n = 11) (B) CDX2 low group tends to express higher CLD18 than CDX2 high group although it is not statistically significant (p = 0.399). (TIF) [file pone.0082390.s004.tif]

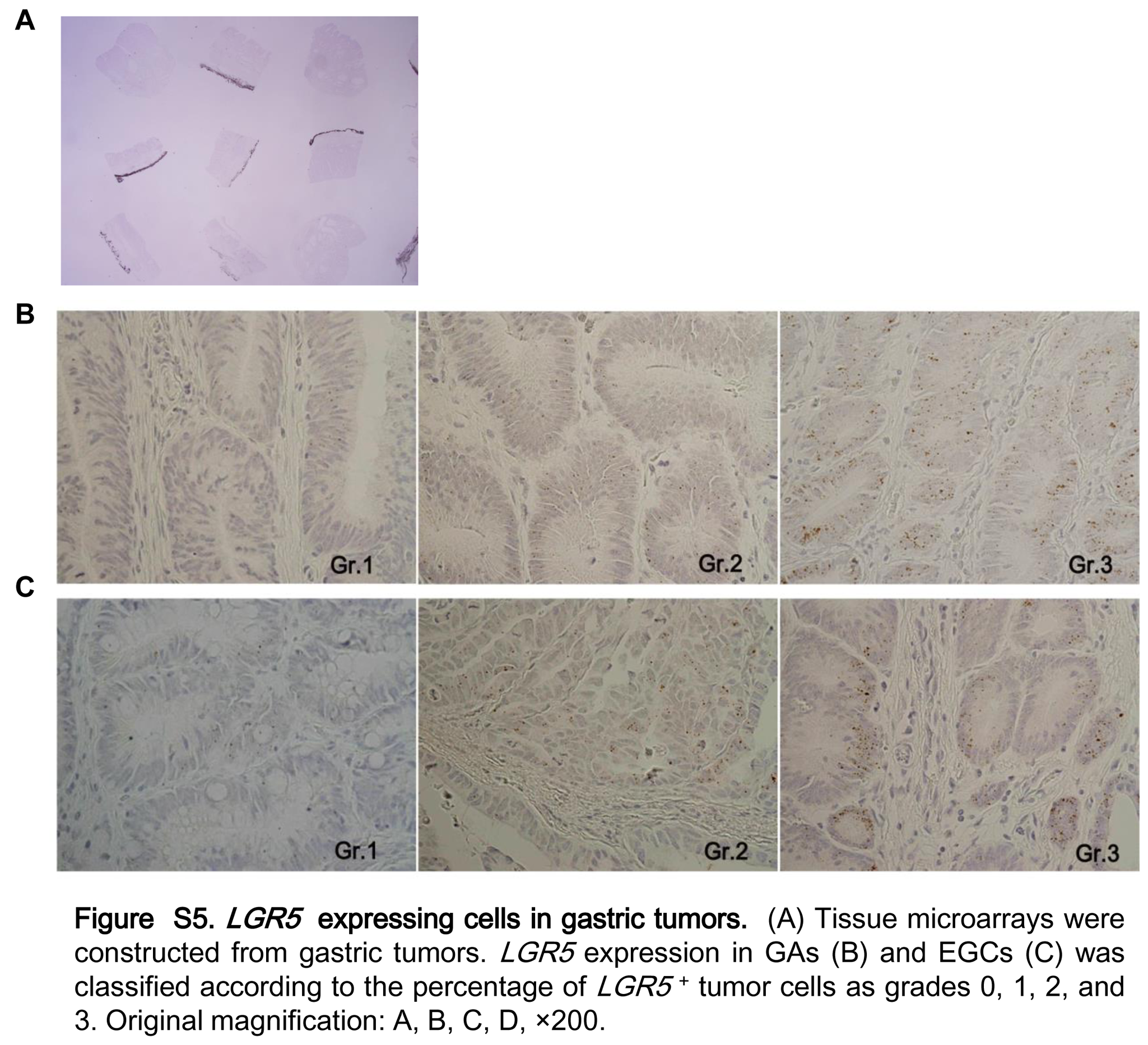

Supplement: Figure S5 — LGR5 expressing cells in gastric tumors. (A) Tissue microarrays were constructed from gastric tumors. LGR5 expression in GAs (B) and EGCs (C) was classified according to the percentage of LGR5 + tumor cells as grades 0, 1, 2, and 3. Magnification: A, B, C, D, ×200. (TIF) [file pone.0082390.s005.tif]

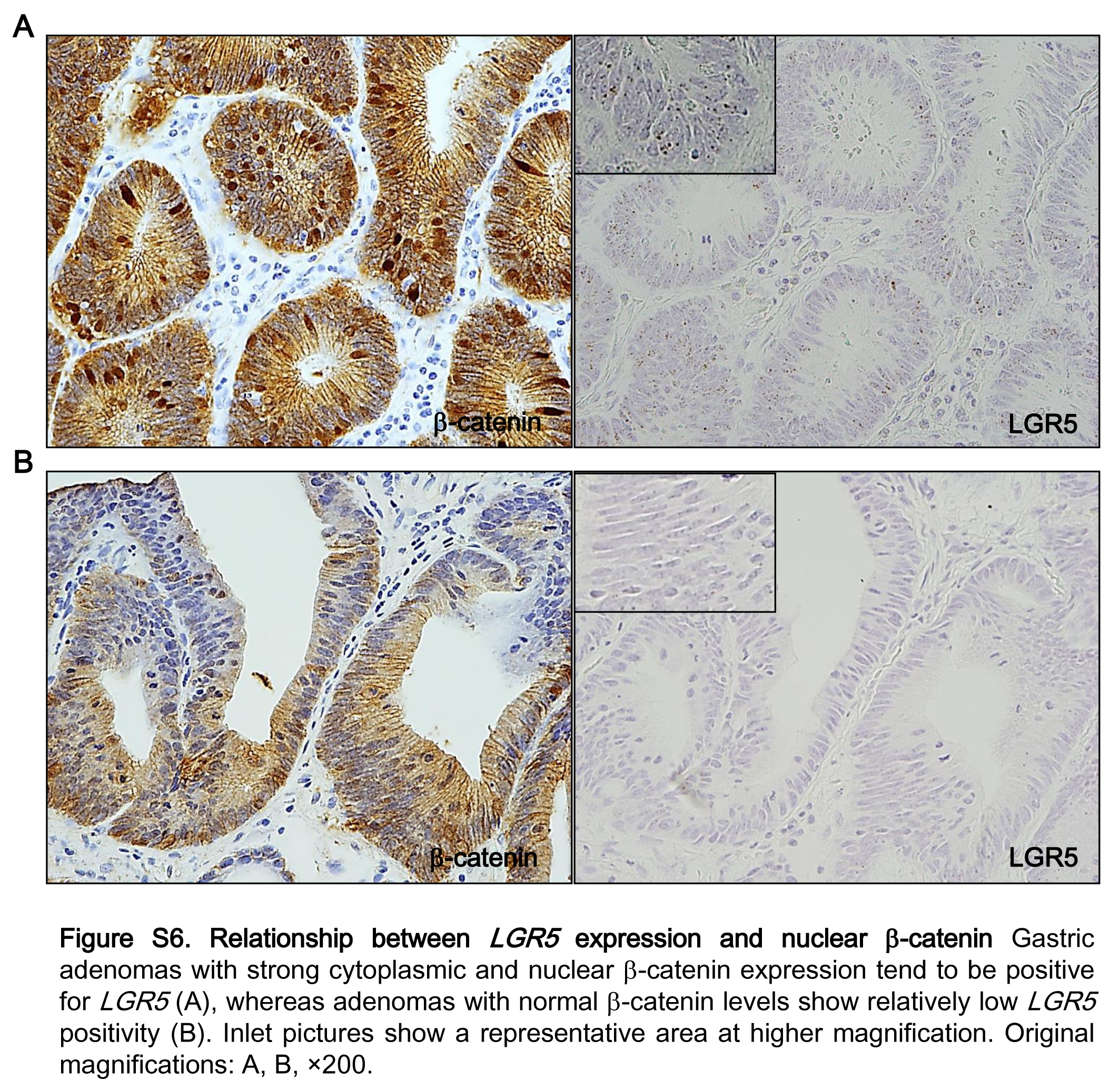

Supplement: Figure S6 — Relationship between LGR5 expression and nuclear β-catenin Gastric adenomas with strong cytoplasmic and nuclear β-catenin expression tend to be positive for LGR5 (A), whereas adenomas with normal β-catenin levels show relatively low LGR5 positivity (B). Inlet pictures show a representative area at higher magnification. Magnifications: A, B, ×200. (TIF) [file pone.0082390.s006.tif]

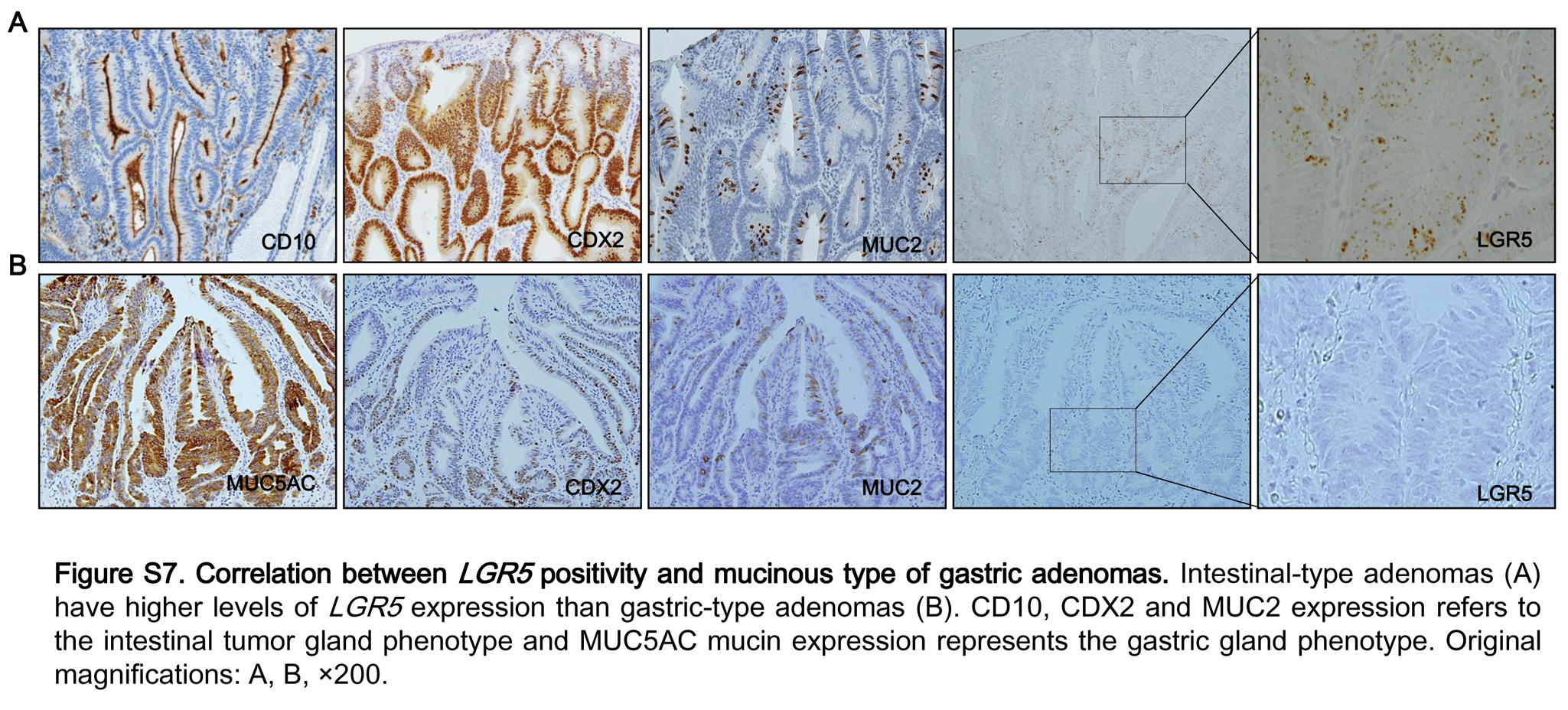

Supplement: Figure S7 — Correlation between LGR5 positivity and mucinous type of gastric adenomas. Intestinal-type adenomas (A) have higher levels of LGR5 expression than gastric-type adenomas (B). CD10, CDX2 and MUC2 expression refers to the intestinal tumor gland phenotype and MUC5AC mucin expression represents the gastric gland phenotype. Magnifications: A, B, ×200. (TIF) [file pone.0082390.s007.tif]

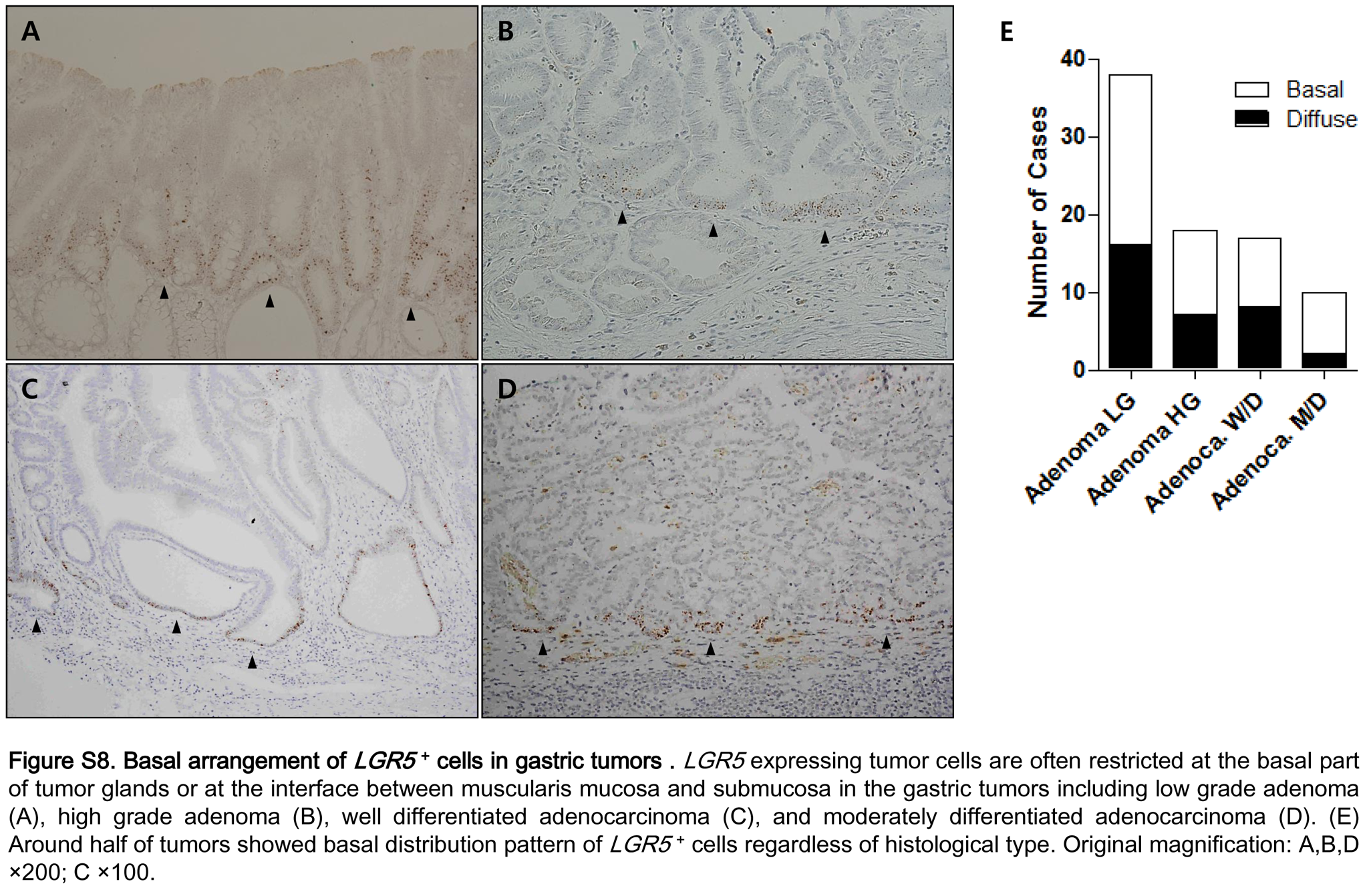

Supplement: Figure S8 — Basal arrangement of LGR5 + cells in gastric tumors. LGR5 expressing tumor cells are often restricted at the basal part of tumor glands or at the interface between muscularis mucosa and submucosa in the gastric tumors including low grade adenoma (A), high grade adenoma (B), well differentiated adenocarcinoma (C), and moderately differentiated adenocarcinoma (D). (E) Around half of tumors showed basal distribution pattern of LGR5 + cells regardless of histological type. Magnification: A, B, D ×200; C ×100. (TIF) [file pone.0082390.s008.tif]

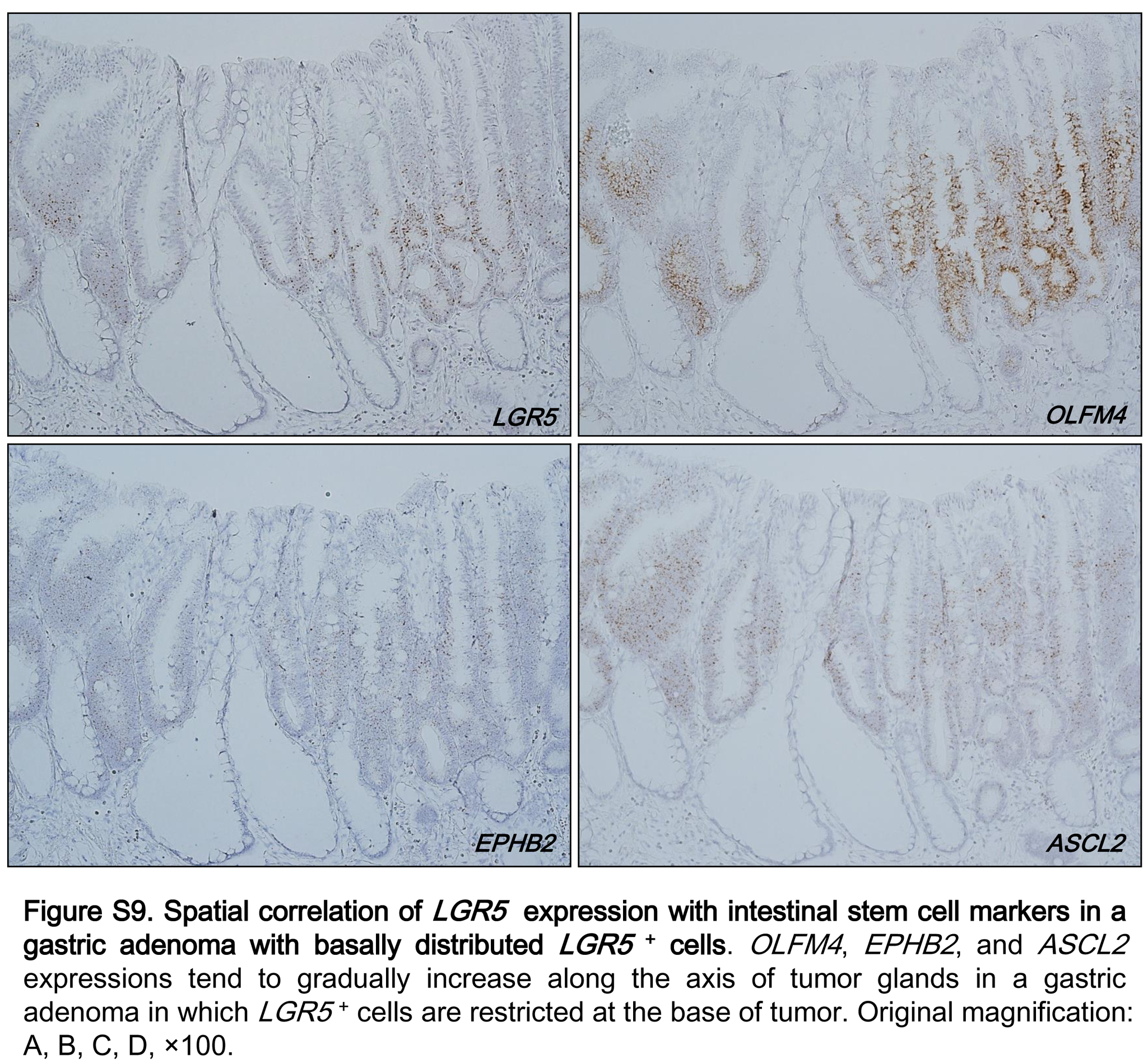

Supplement: Figure S9 — Spatial correlation of LGR5 expression with intestinal stem cell markers in a gastric adenoma with basally distributed LGR5 + cells. OLFM4, EPHB2, and ASCL2 expressions tend to gradually increase along the axis of tumor glands in a gastric adenoma in which LGR5 + cells are restricted at the base of tumor. Magnification: A, B, C, D, ×100. (TIF) [file pone.0082390.s009.tif]

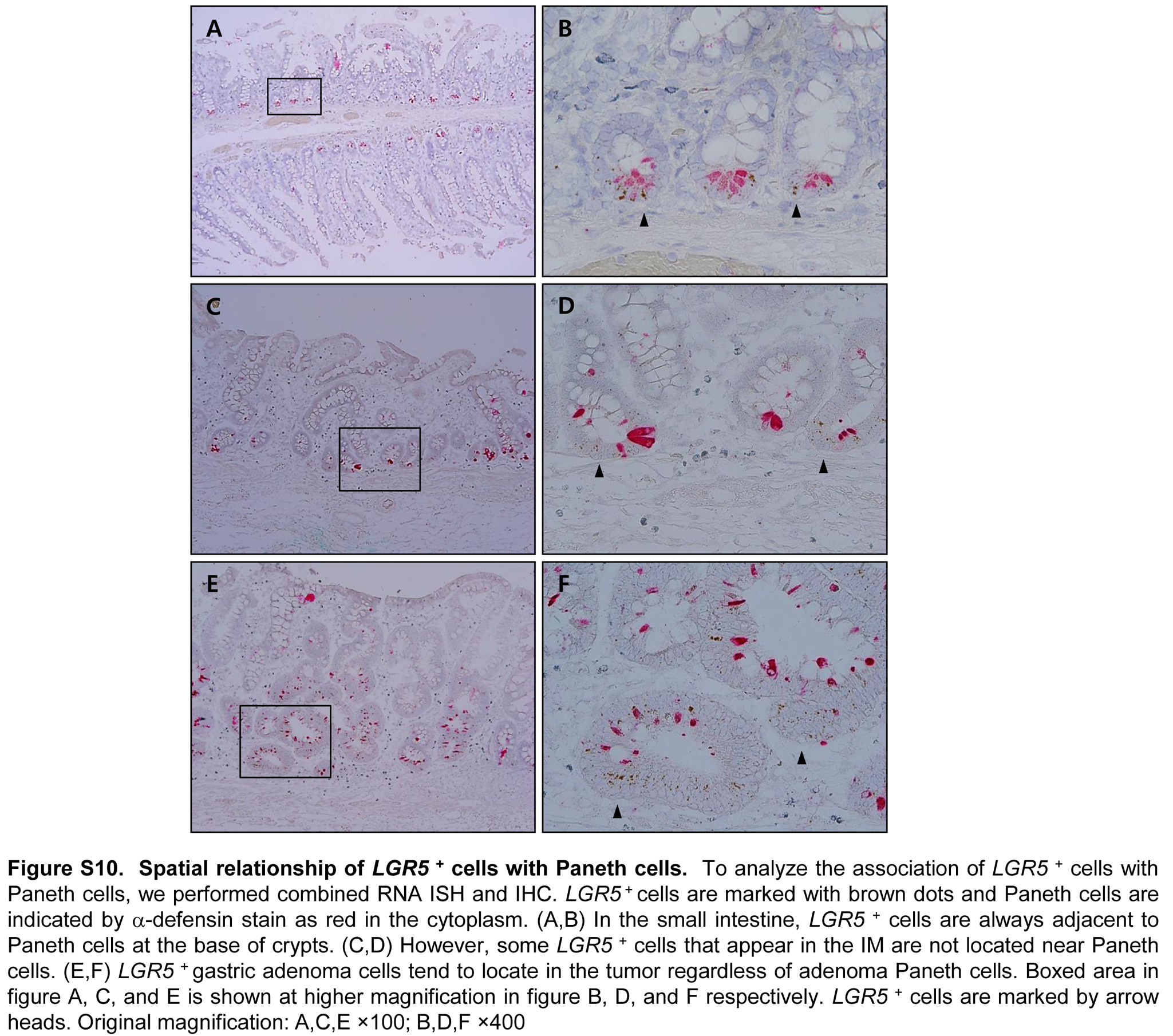

Supplement: Figure S10 — Spatial relationship of LGR5 + cells with regard to Paneth cells. To analyze the association of LGR5 + cells with Paneth cells, we performed combined RNA ISH and IHC. LGR5 + cells are marked with brown dots and Paneth cells are indicated by α-defensin stain as red in the cytoplasm. (A, B) In the small intestine, LGR5 + cells are always adjacent to Paneth cells at the bas β e of crypts. (C, D) However, some LGR5 + cells that appear in the IM are not located near Paneth cells. (E, F) LGR5 + gastric adenoma cells tend to locate in the tumor regardless of adenoma Paneth cells. Boxed areas in figure A, C, and E are shown at higher magnification in figure B, D, and F respectively. LGR5 + cells are marked by arrow heads. Magnification: A, C, E ×100; B, D, F ×400. (TIF) [file pone.0082390.s010.tif]

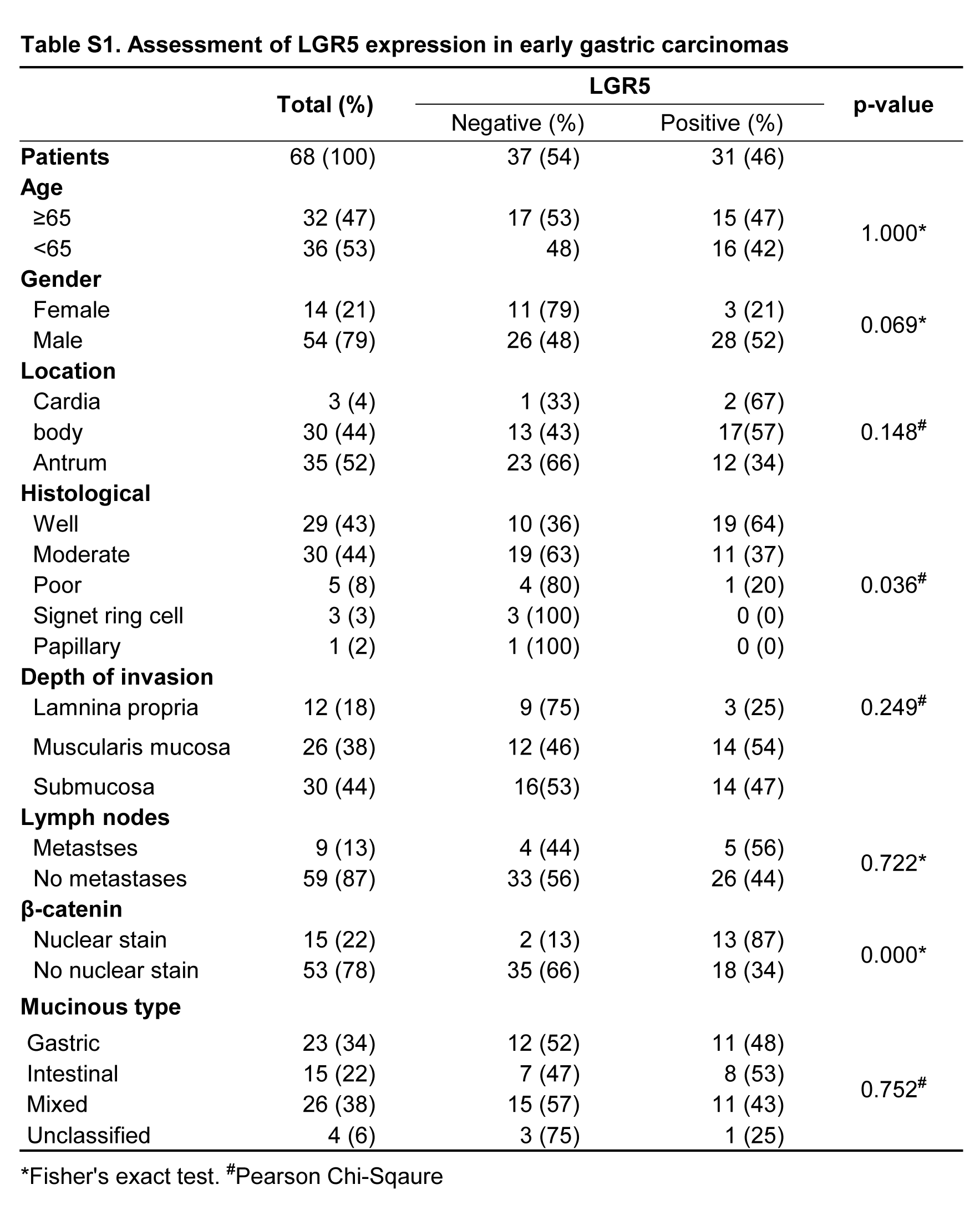

Supplement: Table S1 — Assessment of LGR5 expression in early gastric carcinomas. (TIF) [file pone.0082390.s011.tif]

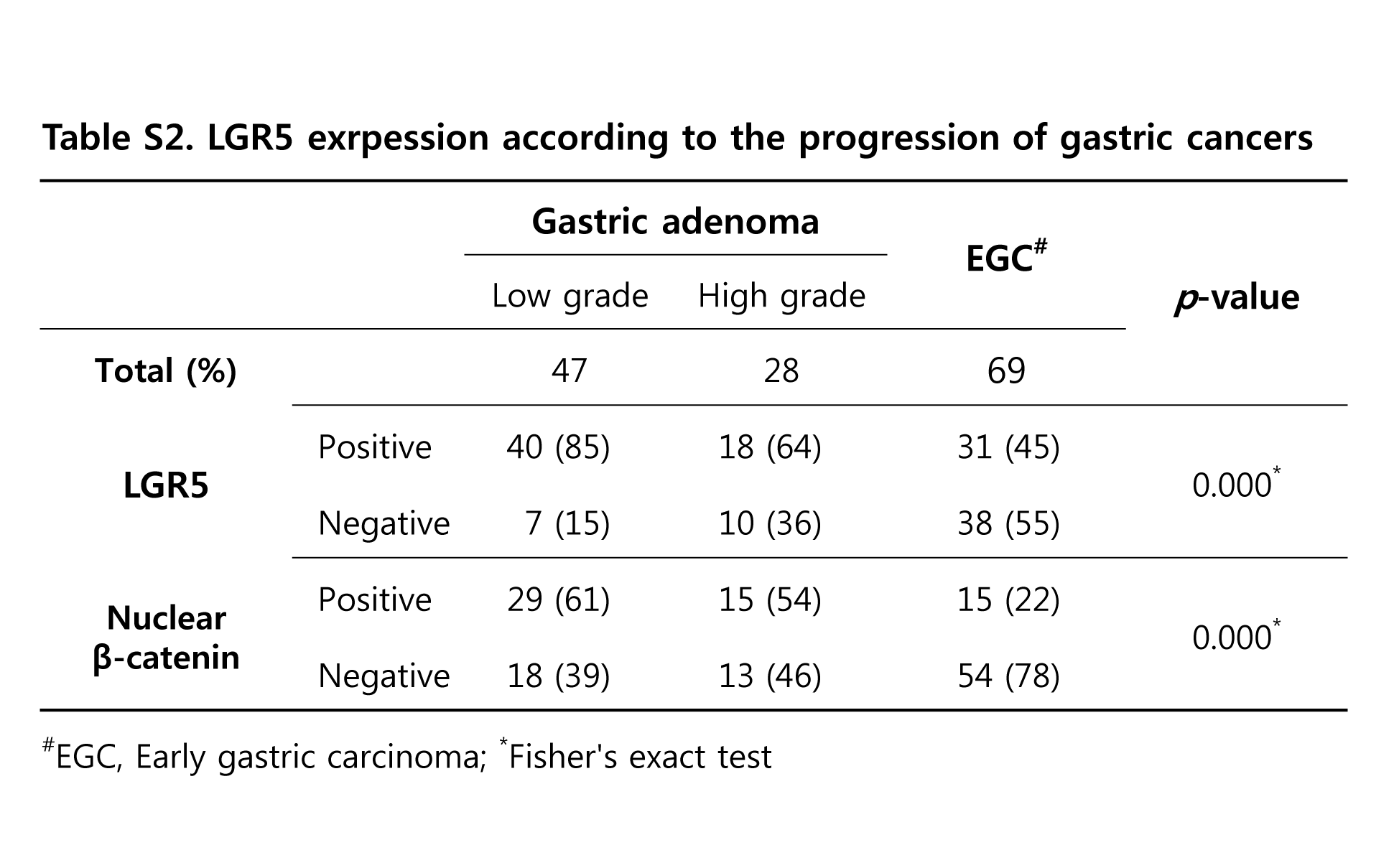

Supplement: Table S2 — LGR5 and nuclear β-catenin with the progression of gastric cancers. (TIF) [file pone.0082390.s012.tif]
